# Supplementary material for: LDL-C:HDL-C ratio and common carotid plaque in Xinjiang Uygur obese adults: a cross-sectional study
Source: BMJ Open. 2018 Oct 8;8(10):e022757. doi: 10.1136/bmjopen-2018-022757 (PMC6194467; doi:10.1136/bmjopen-2018-022757)
Supplement: Author's manuscript [file bmjopen-2018-022757.draft_revisions.pdf]

BMJ Open is committed to open peer review. As part of this commitment we make the peer review history of every article we publish publicly available.

When an article is published we post the peer reviewers' comments and the authors' responses online. We also post the versions of the paper that were used during peer review. These are the versions that the peer review comments apply to.

The versions of the paper that follow are the versions that were submitted during the peer review process. They are not the versions of record or the final published versions. They should not be cited or distributed as the published version of this manuscript.

BMJ Open is an open access journal and the full, final, typeset and author-corrected version of record of the manuscript is available on our site with no access controls, subscription charges or pay-per-view fees (http://bmjopen.bmj.com).

If you have any questions on BMJ Open's open peer review process please email info.bmjopen@bmj.com

BMJ Open

LDL-C/HDL-C ratio contributes to common carotid plaque among obese Uygur adults in Xinjiang, China

Journal:	*BMJ Open*
Manuscript ID	bmjopen-2018-022757
Article Type:	Research
Date Submitted by the Author:	07-Mar-2018
Complete List of Authors:	Zhao, Qiang; First Affiliated Hospital of Xinjiang Medical University, Department of Cardiology<br>Liu, fen; First Affiliated Hospital of Xinjiang Medical University, Department of cardiology<br>Wang, Ying-Hong; First Affiliated Hospital of Xinjiang Medical University, Department of Health Management & Physical Examination Center<br>Lai, Hong-Mei; People's Hospital of Xinjiang Uygur Autonomous Region, Department of Cardiology<br>Zhao, Qian; First Affiliated Hospital of Xinjiang Medical University, Department of Cardiology<br>Luo, Jun-Yi; First Affiliated Hospital of Xinjiang Medical University, Department of Cardiology<br>Ma, Yi-Tong; First Affiliated Hospital of Xinjiang Medical University, Department of Cardiology<br>Li, Xiao-Mei<br>Yang, Yi-Ning; First Affiliated Hospital of Xinjiang Medical University, Department of Cardiology
Keywords:	lipid ratio, carotid atherosclerotic plaque, obesity, Uygur

SCHOLARONE™
Manuscripts

LDL-C/HDL-C ratio contributes to common carotid plaque among obese Uygur adults in Xinjiang, China

Qiang Zhao[1, 2, *], Fen Liu[1, 2, *], Ying-Hong Wang[1, 2, 3], Hong-Mei Lai[4], Qian Zhao[1, 2], Jun-Yi Luo[1, 2], Yi-Tong Ma[1, 2], Xiao-Mei Li[1, 2], Yi-Ning Yang[1, 2]

[1] Department of Cardiology, First Affiliated Hospital of Xinjiang Medical University, Urumqi, China;

[2] Xinjiang Key Laboratory of Cardiovascular Disease Research, Urumqi, China;

[3] Department of Health Management & Physical Examination Center, First Affiliated Hospital of Xinjiang Medical University, Urumqi, China;

[4] Department of Cardiology, People's Hospital of Xinjiang Uygur Autonomous Region, Urumqi, China.

* QZ and FL equally contributed.

Corresponding author: Yi-Ning Yang,

Department of Cardiology, First Affiliated Hospital of Xinjiang Medical University,

137 Liyushan Road, Urumqi, 830054 Xinjiang, China.

Tel: 86-991-4365381, e-mail: yangyn5126@163.com.

Short title: LDL-C/HDL-C ratio and common carotid artery plaque

Word count: 2934 Abstract: 240 Tables:4 Figures: 2

For peer review only - http://bmjopen.bmj.com/site/about/guidelines.xhtml

Abstract

Objective The aim of this study was to investigate the relationship between LDL-C/HDL-C ratio and common carotid atherosclerotic plaque (CCAP) among Uygur obese adults in Xinjiang, China.

Design Cross-sectional study. Participants completed the lipid profiles and carotid ultrasonography.

Setting General and teaching hospital in the provincial capital of Xinjiang, China.

Participants A total of 1449 obese Uygur adults free of clinical coronary artery disease was enrolled.

Main outcome measures The LDL-C/HDL-C ratio was calculated, and the CCAP measured by ultrasound was identified. Logistic regression analyses were used to assess associations between risk factors and CCAP, and the receiver operating characteristics (ROC) curve was performed to detect the cutoff value of LDL-C/HDL-C ratio for predicting CCAP.

Results Four hundred and fifteen (28.6%) participants had CCAP. The patients with CCAP had significantly higher LDL-C/HDL-C ratio in comparison with their participants without CCAP ($P < 0.001$). In logistic regression model, BMI, smoking, diabetes, LDL-C, and LDL-C/HDL-C ratio were independent predictors of CCAP after adjusting for conventional cardiovascular risk factors. In a ROC analysis, LDL-C/HDL-C ratio > 2.86 determined CCAP with 64.3 % sensitivity and 72.1 % specificity.

Conclusions An increased LDL-C/HDL-C ratio was an independent predictor of CCAP which was highly prevalent among Uygur obese adults. It may help to identify obese population requiring more aggressive LDL-lowering therapy.

Keywords: lipid ratio; carotid atherosclerotic plaque; obesity; Uygur

For peer review only

For peer review only - http://bmjopen.bmj.com/site/about/guidelines.xhtml

Strengths and limitations of this study

1. This is an in-depth investigation of the associations between the LDL-C/HDL-C ratio and common carotid atherosclerotic plaque in a Chinese Uygur population.

2. The present study identify that the cutoff value of LDL-C/HDL-C ratio for determining the carotid plaque was 2.86 for Uygur population.

3. The cross-sectional design does not confirm the causality between LDL-C/HDL-C ratio and carotid plaque risk.

4. Potential bias might exist in a single-center design.

For peer review only

For peer review only - http://bmjopen.bmj.com/site/about/guidelines.xhtml

1. Introduction

The prevalence of obesity has been increasing in China during the past few decades. Nowadays, people who are overweight or obese account for one thirds of Chinese adults. [1 2] Cardiometabolic risk factors including hypertension, dyslipidemia and type 2 diabetes were more prevalent in obese adults, which partly explained the association between obesity and increased cardiovascular disease (CVD). [3-5] Recent data demonstrated that obese individuals who were even metabolically healthy had a higher risk of CVD including coronary artery disease (CAD), heart failure, and cerebrovascular disease than normal weight metabolically healthy individuals. [6] Obesity has been identified as an independent risk factor for CVD and death, [3 7] and it is emerging as major public health challenges in China.

Atherosclerosis (AS) is the primary cause for mortality and morbidity in CVD. [8] It is well known that AS is a systemic process which begins early in childhood and typically remains asymptomatic until later in life. [9] Approximately 30% of first acute events are fatal, so the prevention of CVD should be directed towards the risk factors and the silent preclinical phase of the process. As a non-invasive ultrasound biomarker of AS, the carotid intima-media thickness (CIMT) has been demonstrated as a reliable surrogate of risk of cardiac events. [10 11] Of all the segments of the carotid artery which have been used in CIMT, the common carotid artery (CCA) is the easiest to image and has the highest reproducibility. Prior researches have described that the presence of common carotid atherosclerotic plaque (CCAP) was correlated with the increased prevalence of CAD, myocardial infarction, cardiac mortality and stroke beyond traditional cardiovascular risk factors. [10 12-14] Thus, early detection and treatment initiation of patients with CCAP in the obesity might be essential for improvement in the prevention of cardiovascular events.

Many cohort and nested case-control studies showed obesity is closely related with high prevalence of dyslipidemia, characterized by increased plasma triglycerides (TG), total cholesterol (TC), and low-density lipoprotein cholesterol (LDL-C), together with lower high-density lipoprotein cholesterol (HDL-C) levels. [15-18] Previous studies have established the important role of LDL-C in the pathogenesis of AS. LDL-C has been used to monitor the lipid-lowering response to treatments in almost all patients with CVD. However, a number of clinical trials showed 60 - 70 % of CVD events continue to occur despite LDL-lowering

For peer review only - http://bmjopen.bmj.com/site/about/guidelines.xhtml

therapy, which may be due to residual risks associated with lipid abnormalities especially atherogenic dyslipidemia, including increased TG levels and reduced HDL-C levels. [19 20] Therefore, a need for new targets to compliment the measures of LDL-C lowering has been suggested. Recent studies identified that LDL-C/HDL-C ratio as a better biomarker to predict AS progression and CVD than LDL-C or HDL-C alone. [21-24] However, few studies have investigated whether LDL-C/HDL-C ratio was associated with CCAP among obese population. Thus, the present study was designed to explore the relationship between the level of LDL-C/HDL-C ratio and CCAP among Uygur obese adults in Xinjiang.

2. Subjects and methods

Study design and participants

The present study was a cross-sectional study undertook at the First Affiliated Hospital of Xinjiang Medical University from 1 January 2014 to 31 December 2016.

The study population was enrolled from the outpatient clinic and inpatient department of cardiology as well as department of physical examination center at the First Affiliated Hospital of Xinjiang Medical University. All subjects were obese Uygur participants free of clinically evident CAD, defined as a previous myocardial infarction, previous or current angina with objective evidence of atherosclerotic CAD, or a previous coronary revascularization procedure. Exclusion criteria for the present analysis were a history of CAD, stenosis of carotid artery＞30%, transient ischemic attack or stroke, current cancer, chronic kidney or liver diseases, and thyroid diseases.

Ethics statement

The Ethics Committee of the Faculty of Medicine at First Affiliated Hospital of Xinjiang Medical University approved this study. Appropriate written informed consents were obtained from all subjects.

Risk factors and definitions

Medical history was obtained and physical examination was performed in all participants. The measured atherosclerosis risk factors relevant to our study were hypertension, diabetes mullites, cigarette smoking status, lipid profiles and body mass index (BMI).

Hypertension was defined as history of hypertension and/or an average systolic blood

For peer review only - http://bmjopen.bmj.com/site/about/guidelines.xhtml

pressure ≥ 140 mmHg and/or an average diastolic blood pressure ≥ 90 mmHg. [25] The presence of diabetes mellitus was based on a history of diabetes, defined as either a current or previous fasting plasma glucose level of 7 mmol/L or higher or current or when participants were taking antihypertensive medication. [26] BMI was calculated by dividing the weight in kilograms by the height in meters squared. Obesity was defined as BMI ≥ 28.0 kg/m^2 by Chinese standard. [2] Current smoking was defined as smoking in the present or having smoked in the previous 6 months.

Lipid profile analyses

Fasting blood samples were collected from all subjects for the assessment of routine laboratory parameters. Fasting plasma glucose, blood urea nitrogen, creatinine, and uric acid were determined by the standard methods. All lipid analyses, including TG, TC, LDL-C, and HDL-C were performed at the Core Laboratory of the First Affiliated Hospital of Xinjiang Medical University using standard enzymatic methods. Dyslipidemia was defined as having at least one of the following conditions: TC concentration > 6.22 mmol/L, LDL-C concentration > 4.14 mmol/L, HDL-C concentration < 1.04 mmol/L, TG concentration > 2.26 mmol/L, or self-reported use of lipid-lowering drugs. [27]

Carotid ultrasonography assessments

The Acuson Sequoia 512 instrument ultrasound mainframes with a 7.5-Mhz linear array transducer (Siemens AG, Erlangen, Germany) was used to acquire ultrasonographic images of both the right and left CCAs. Intima–media interface lines were manually traced as continuous lines by certified sonographers. The IMT was measured over a segment of the CCA that was 1 cm long, located approximately 0.5 cm below the carotid-artery bulb, and considered not to contain any plaque and measured as the distance between the media-adventitia and intima-lumen interfaces [26]. We performed three views on each side of CCAs to determine the maximal and mean CIMT. The presence of CCAP was defined as a CIMT > 1.5 mm, or a focal structure encroaching into the arterial lumen of at least 0.5 mm, or 50% of the surrounding CIMT value. [28 29] Carotid ultrasonography was performed and evaluated by two independent ultrasonologists who were masked to the clinical and laboratory data of all participants. If the diagnostic results from above two ultrasonologists were inconsistent, a third ultrasonologist was obtained. Consensus was achieved in all patients.

For peer review only - http://bmjopen.bmj.com/site/about/guidelines.xhtml

Statistical analysis

Statistical analyses were conducted using the SPSS version 17.0 (SPSS Inc., Chicago, Illinois). Normally distributed continuous data were presented as mean ± standard deviation and compared using unpaired t-tests, while non-normally distributed data were summarized as median with interquartile range (IQR) and compared using Mann-Whitney tests. Categorical variables were summarized in numbers and percentages and compared between groups with Chi-square tests. Univariate logistic regression model was used to estimate the independent predictors of CCAP. After performing univariate analysis, significantly obtained variables (age, BMI, smoking, diabetes, TC, LDL-C, HDL-C together with LDL-C/HDL-C ratio) were used in multivariate logistic regression analysis. The receiver operating characteristics (ROC) curve was applied to demonstrate the sensitivity and specificity of LDL-C/HDL-C ratio for determining CCAP. Additionally, the distance on the ROC curve of each LDL-C/HDL-C ratio was calculated as the square root of $[(1 - \text{sensitivity})^2 + (1 - \text{specificity})^2]$, and the ratio with the shortest distance on the ROC curve was considered as the optimal cutoff value. [28 29] A 2-tailed value of $P < 0.05$ was considered significant.

3. Results

A total of 1449 participants with a mean age of 53.81 ± 11.52 and no history of CAD were recruited in this study. There are 415 (28.6 %) participants in the CCAP group (mean age 55.29 ± 11.04 years and 54.0 % men) and 1034 participants (71.4 %) in the non-CCAP group (mean age 53.22 ± 11.67 years and 51.9 % men).

Baseline characteristics of the study participants

The baseline characteristics of the patients stratified by the presence of CCAP are demonstrated in Table 1. The two groups did not differ with respect to sex ratio, systolic blood pressure, diastolic blood pressure, the prevalence of hypertension or previous medications. The participants in the CCAP group were more likely to be older with higher BMI, and higher prevalence of diabetes and smoking than those in the non-CCAP group (All $P < 0.05$).

Table 1. Baseline characteristics of study population

Characteristics	Overall	CCAP group	Non-CCAP group	*P*-value

For peer review only - http://bmjopen.bmj.com/site/about/guidelines.xhtml

	(n = 1449)	(n = 415)	(n = 1034)	
Demographics				
Male sex, n (%)	761 (52.5)	224 (54.0)	537 (51.9)	0.482
Age, years	53.81 ± 11.52	55.29 ± 11.04	53.22 ± 11.67	0.002
Vital signs				
Body mass index, kg/m^2	30.12 (28.90, 32.32)	30.39 (29.06, 32.74)	30.10 (28.76, 32.01)	0.027
Systolic blood pressure, mmHg	130.19 ± 14.13	130.99 ± 12.94	129.88 ± 14.58	0.176
Diastolic blood pressure, mmHg	80.98 ± 8.42	80.67 ± 8.10	81.11 ± 8.54	0.369
Medical histories				
Smoking, n (%)	246 (17.0)	90 (21.7)	156 (15.1)	0.002
Hypertension, n (%)	444 (30.6)	123 (29.6)	323 (31.0)	0.600
Diabetes mellitus, n (%)	324 (22.4)	133 (32.0)	191 (18.5)	< 0.001
Previous Medications				
Antihypertensive drugs, n (%)	311 (21.5)	83 (22.0)	228 (22.1)	0.390
Antidiabetic agents, n (%)	147 (10.1)	51 (12.3)	96 (9.3)	0.087
Statins, n (%)	146 (10.1)	37 (8.9)	109 (10.5)	0.353

CCAP, common carotid atherosclerotic plaque.

Laboratory findings are summarized in Table 2. Of 1449 participants, 48.2 % of them had dyslipidemia. No significant differences were observed in the plasma levels of TG, uric acid, blood urea nitrogen, creatinine, and transaminases between the two groups. Participants with CCAP had significantly higher plasma concentrations of fasting plasma glucose, TC, and LDL-C, and higher prevalence of dyslipidemia as well as lower HDL-C level than those in the non-CCAP group. Furthermore, the LDL-C/HDL-C ratio of patients with CAAP was significantly higher than those without CCAP (Fig 1).

Table 2. Laboratory and ultrasonographic measurements of study groups

Characteristics	Overall (n = 1449)	CCAP group (n = 415)	Non-CCAP group (n = 1034)	*P*-value

For peer review only - http://bmjopen.bmj.com/site/about/guidelines.xhtml

Laboratory measurements				
White blood cell count, 10^9/L	6.82 ± 1.87	6.71 ± 1.92	6.87 ± 1.86	0.147
Platelet count, 10^9/L	212.13 ± 50.79	213.45 ± 51.19	211.60 ± 50.65	0.531
Hemoglobin, g/L	137.02 ± 15.71	136.49 ± 15.42	137.24 ± 15.82	0.408
Fasting plasma glucose, mmol/L	5.28 (4.66, 5.93)	5.73 (5.17, 6.48)	5.08 (4.59, 5.65)	< 0.001
TG, mmol/L	1.54 ± 0.86	1.51 ± 0.85	1.56 ± 0.87	0.327
TC, mmol/L	4.89 ± 0.87	5.11 ± 0.84	4.80 ± 0.87	< 0.001
LDL-C, mmol/L	3.04 ± 0.66	3.44 ± 0.69	2.88 ± 0.58	< 0.001
HDL-C, mmol/L	1.16 (1.00, 1.35)	1.10 (0.92, 1.28)	1.19 (1.03, 1.38)	< 0.001
LDL-C/HDL-C ratio	2.52 (2.07, 3.25)	3.21 (2.50, 3.88)	2.33 (1.95, 2.97)	< 0.001
Dyslipidemia, n (%)	698 (48.2)	267 (64.3)	431 (41.7)	< 0.001
Blood urea nitrogen, mmol/L	4.87 (4.10, 5.80)	4.97 (4.10, 5.90)	4.80 (4.10, 5.71)	0.284
Creatinine, μmol/L	70.90 ± 15.34	71.82 ± 15.68	70.54 ± 15.19	0.150
Uric acid, mg/dL	5.16 ± 1.40	5.23 ± 1.43	5.13 ± 1.39	0.214
Glutamic-pyruvic transaminase, U/L	27.56 ± 15.64	28.44 ± 16.15	27.21 ± 15.42	0.179
Glutamic-oxaloacetic transaminase, U/L	19.80 (16.50, 24.21)	19.60 (16.60, 24.20)	19.80 (16.50, 24.23)	0.850
Ultrasonographic parameters				
Maximal CCA-IMT, mm	1.10 (0.80, 1.40)	1.60 (1.50, 1.80)	0.90 (0.70, 1.10)	< 0.001
Mean CCA-IMT, mm	0.80 (0.60, 1.10)	1.30 (1.10, 1.40)	0.80 (0.60, 0.90)	< 0.001
Unilateral plaque, n (%)	227 (54.7)	227 (54.7)	-	-
Bilateral plaque, n (%)	188 (45.3)	188 (45.3)	-	-
Maximum CCA-IMT > 0.9 mm, n (%)	429 (41.5)	-	429 (41.5)	-

TG, triglyceride; TC, total cholesterol; LDL-C, low density lipoprotein-cholesterol; HDL-C, high density lipoprotein-cholesterol; CCA-IMT, common carotid artery intima–media thickness.

The carotid ultrasound examinations of the participants are also presented in Table 2. Of 415 participants in the CCAP group, 54.7 % participants had unilateral plaque, and 45.3 %

For peer review only - http://bmjopen.bmj.com/site/about/guidelines.xhtml

participants had bilateral plaques. The maximal and mean CCA-IMT were 1.60 mm (IQR: 1.50 - 1.80) and 1.30 mm (IQR: 1.10 - 1.40) in the CCAP group, respectively. In non-CCAP group, 41.5 % participants had maximum CCA-IMT higher than 0.9mm, which represented carotid intimal thickening, and the maximal and mean CCA-IMT were 0.90 mm (IQR: 0.70 - 1.10) and 0.80 mm (IQR: 0.60 - 0.90), respectively. Participants in the CCAP group had higher maximal and mean CCA-IMT than those in non-CCAP group (All $P < 0.001$).

Association of LDL-C/HDL-C ratio and CCAP

Univariate and multivariate logistic regression models of the associations between LDL-C/HDL-C ratio and CCAP are presented in Table 3. In multivariate analyses, Age > 45 years (every 10 years，All $P < 0.05$), BMI (OR: 1.063, 95 % CI: 1.014 - 1.115, $P = 0.011$), smoking (OR: 1.535, 95 % CI: 1.097 - 2.147, $P = 0.012$), diabetes (OR: 1.526, 95 % CI: 1.135 - 2.052, $P = 0.005$), LDL-C (OR: 2.127, 95 % CI: 1.258 - 3.594, $P = 0.005$), and LDL-C/HDL-C ratio (OR: 2.468, 95 % CI: 1.473 - 4.136, $P = 0.001$) were independent predictors of CCAP after adjustments for traditional cardiovascular risk factors, including age, BMI, smoking, and diabetes.

Table 3. Logistic regression models for variables associated with CCAP

Parameters	Univariate			Multivariate		
	OR	95 % CI	*P*-value	OR	95 % CI	*P*-value
Male sex	1.085	0.864 - 1.364	0.482	NI		
Age			0.013			0.001
<45 years	1			1		
45 - 54 years	1.631	1.178 - 2.257	0.003	1.531	1.056 - 2.220	0.025
55 - 64 years	1.485	1.048 - 2.104	0.026	2.069	1.390 - 3.079	< 0.001
65 - 74 years	1.625	1.132 - 2.333	0.009	1.931	1.270 - 2.937	0.002
≥ 75 years	2.288	1.220 - 4.292	0.010	3.352	1.635 - 6.872	0.001
Body mass index	1.068	1.024 - 1.114	0.002	1.063	1.014 - 1.115	0.011
Smoking	1.559	1.167 - 2.081	0.003	1.535	1.097 - 2.147	0.012
Hypertension	0.936	0.730 - 1.200	0.600	NI		
Diabetes mellitus	2.082	1.606 - 2.697	< 0.001	1.526	1.135 - 2.052	0.005

For peer review only - http://bmjopen.bmj.com/site/about/guidelines.xhtml

TG	0.935	0.817 - 1.070	0.327	NI		
TC	1.512	1.323 - 1.728	< 0.001	0.963	0.771 - 1.203	0.738
LDL-C	3.012	2.274 - 3.917	< 0.001	2.127	1.258 - 3.594	0.005
HDL-C	0.222	0.145 - 0.340	< 0.001	1.574	0.453 - 3.464	0.475
LDL-C/HDL-C ratio	3.156	2.692 - 3.700	< 0.001	2.468	1.473 - 4.136	0.001
Uric acid	1.053	0.971 - 1.141	0.214	NI		
Antihypertensive drugs	0.884	0.667 - 1.172	0.390	NI		
Antidiabetic agents	1.369	0.955 - 1.963	0.088	NI		
Statins	0.831	0.561 - 1.229	0.353	NI		

NI, not included in the model.

For peer review only

The ROC analysis of LDL-C/HDL-C ratio for determining CCAP is presented in Fig 2. In the present study, LDL-C/HDL-C ratio was closely related with the presence of CCAP among obese Uygur population. The area under the ROC curve for LDL-C/HDL-C ratio was 0.748 (95 % CI: 0.720 - 0.776, P < 0.001), and a ratio of 2.86 was identified as the appropriate cutoff value and determined CCAP with a sensitivity of 64.3 % and a specificity of 72.1 %.

When we divided all patients into two groups based on the cutoff value of LDL-C/HDL-C ratio identified in ROC analysis, participants with LDL-C/HDL-C ratio > 2.86 had higher prevalence of CCAP (47.8 % vs. 16.6 %, P<0.001), diabetes (33.9 % vs. 15.2 %, P<0.001), and dyslipidemia (80.3 % vs. 28.1 %, P<0.001) than their participants with LDL-C/HDL-C ratio ≤ 2.86. Meanwhile, the maximal and mean CCA - IMT was also significantly higher in participants with LDL-C/HDL-C ratio > 2.86 (Table 4).

Table 4. Clinical characteristics of study population according to LDL-C/HDL-C ratio cutoff

Characteristics	LDL-C/HDL-C ratio > 2.86 (n = 558)	LDL-C/HDL-C ratio ≤ 2.86 (n = 891)	*P*-value
Male sex, n (%)	301 (53.9)	450 (51.6)	0.390
Age, years	53.69 ± 11.24	53.89 ± 11.70	0.751
Body mass index, kg/m^2	30.28 (29.01, 32.70)	30.10 (28.73, 32.01)	0.034

For peer review only - http://bmjopen.bmj.com/site/about/guidelines.xhtml

Smoking, n (%)	122 (21.9)	124 (13.9)	< 0.001
Hypertension, n (%)	177 (31.7)	267 (30.0)	0.481
Antihypertensive drugs, n (%)	121 (21.7)	190 (21.3)	0.871
Diabetes mellitus, n (%)	189 (33.9)	135 (15.2)	< 0.001
Antidiabetic agents, n (%)	64 (11.5)	83 (9.3)	0.186
Dyslipidemia, n (%)	448 (80.3)	250 (28.1)	< 0.001
Statins, n (%)	50 (9.0)	96 (10.8)	0.264
CCAP, n (%)	267 (47.8)	148 (16.6)	< 0.001
Maximal CCA-IMT, mm	1.20 (0.80, 1.60)	1.00 (0.80, 1.20)	< 0.001
Mean CCA-IMT, mm	0.90 (0.70, 1.20)	0.80 (0.60, 1.00)	< 0.001

4. Discussion

In the present study, we examined the associations between LDL-C/HDL-C ratio and CCAP in obese Uygur population. The main findings from our study are as follows: CCAP is highly prevalent in Uygur obesity adults; LDL-C/HDL-C ratio is independently related with CCAP and that the ratio of 2.86 might serve as a cutoff value to increase the prevalence of CCAP among obese Uygur population in Xinjiang.

It is well known that obesity is associated with several cardiovascular risk factors, such as dyslipidemia, type 2 diabetes as well as hypertension, and that all of these are related to increased cardiovascular risks. Recent studies have reported that obesity increased the risk of incident subclinical carotid AS. [30 31] In the present study, 28.6 % obese participants had CCAP, and the best predictive marker of CCAP was LDL-C/HDL-C ratio, and the plasma level of LDL-C and HDL-C also discriminated CCAP. The demonstrated high prevalence of CCAP in obesity individuals expands findings in previous population-based studies in which the association of obesity with atherosclerotic progression has been proven. [31-33]

The causal relationship between LDL-C and increased risk of CAD has been well established. However, even aggressive reduction in LDL-C with statin treatment still leaves considerable residual risk of CAD events. Since from analysis of 2045 patients in the placebo

For peer review only - http://bmjopen.bmj.com/site/about/guidelines.xhtml

group of the Helsinki Heart Study demonstrated the LDL-C/HDL-C ratio was the best single predictor of cardiac events, [34] the LDL-C/HDL-C ratio has recently been considered as a novel predictive marker for AS diseases**.** Previous studies have reported that the elevated LDL-C/HDL-C ratio is related with an increased risk of sudden cardiac death and the progression of both low-grade and high-grade coronary artery lesions. [24 35] Recently, several investigations have examined the utility of LDL-C/HDL-C ratio in predicting carotid AS. However, so far contrasting results have been demonstrated. Enomoto M et al [21] conducted a periodic epidemiological survey to investigate the relationship between lipid profiles and the progression of common carotid IMT, during an 8-year follow up survey, they found that LDL-C/HDL-C ratio was better in predicting IMT progression than HDL-C or LDL-C alone. Katakami N et al [22] explored the relationships between various lipid parameters including lipid ratios and carotid AS in 934 type 2 diabetic patients without obvious atherosclerotic diseases in Japan, they reported that LDL-C/HDL-C ratio is positively corelated with the carotid atherosclerotic plaques. However, Nimkuntod P et al [36] failed to identify the associations between LDL-C/HDL-C ratio with early atherosclerosis CIMT carotid plaques as well as carotid plaques types. The sample of subjects in the study carried out by Nimkuntod P was small may possibly explain for this discrepancy. In our study, LDL-C/HDL-C ratio and the single parameter of lipid profiles including Tg, TC, LDL-C and HDL-C have been analysed in 1449 obese Uygur individuals. We found that individuals with CCAP had significantly higher prevalence of dyslipidemia. Several pathophysiological processes have been proposed to explain the mechanisms of dyslipidemia in obesity population, mainly driven by the increased lipid production and hydrolysis of TG. Higher plasma level of free fatty acid also contributes to dyslipidemia. According to our findings, we found LDL-C/HDL-C ratio is the strongest predictors of CCAP after adjusting for major risk factors, indicating that the aforementioned ratio may influence the progression of CCAP through pathways beyond traditional risk factors. Our finding is consistent with previous studies and proven the association between LDL-C/HDL-C ratio and CCAP in Uygur obese population. We speculate two factors that explain the superiority of LDL-C/HDL-C ratio to either individual LDL-C or HDL-C. First, LDL-C/HDL-C ratio represents the proportion between the atherogenic and anti-atherosclerotic lipoproteins, which has greater predictive value for

For peer review only - http://bmjopen.bmj.com/site/about/guidelines.xhtml

atherosclerotic change. Second, LDL-C/HDL-C ratio was closely correlated with the distribution of HDL subclasses. With increase of the LDL-C/HDL-C ratio, there was a general shift toward smaller-sized HDL particles, implying that the maturation process of HDL was blocked, which may contribute to the progression of AS. [37]

Our previous work described that the cutoff value of LDL-C/HDL-C ratio for assessing CVD risk factors was 2.5 among Uygur adults. [38] In the present study, we obtained that the cutoff value of LDL-C/HDL-C ratio for predicting CCAP was 2.86, which was higher than that of obtained by Kurabayashi M et al, [39] they found that the cutoff value of LDL-C/HDL-C ratio for initiating second prevention of AS was > 1.5, and the cutoff ratio for initiating primary prevention of AS was > 2.0. The cutoff value identified in the present study was also higher than that obtained by Enomoto M et al, [21] they showed that the cutoff value of LDL-C/HDL-C ratio for predicting the progression of IMT was 2.3. The cutoff ratio obtained from our study is inconsistent with those previous studies. may arise because of the racial and ethnic differences. Several reasons may apply to explain these discordances between above studies. First, the participants recruited into our study were obese individuals, the mean level of LDL-C is significantly higher than those previous studies, which may help to explain why the cutoff value of LDL-C/HDL-C ratio in predicting the CCAP was higher in our study. Second, the distinct ethnic origin, diet, life styles, living habits and living environments that are specific to Uygur population may contribute to these discrepancies.

Several limitations of this study should be considered. First, this is a cross-sectional analysis, which limits the temporal interpretation of predictive measurements. Second, the lipid profiles were measured only once, and the relations between lipid ratio changes and outcomes could not be ascertained. Third, the absence of a health control group was the main limitation of our study. Whether our results can be applied to general obese population remains to be investigated. Fourth, statins are known to affect the changes in LDL-C/HDL-C ratio. [40] About 10% participants take statin medication in our study, so we can't evaluate its influence on lipid ratio changes and the progression of CCAP.

In summary, our study demonstrated that LDL-C/HDL-C ratio of > 2.86 was strongly associated with increased risk of CCAP among Uygur obese adults in Xinjiang, China. This suggests that LDL-C/HDL-C ratio might be used as an easy, useful and noninvasive measure

For peer review only - http://bmjopen.bmj.com/site/about/guidelines.xhtml

to identify obese population requiring more aggressive LDL-lowering therapy. Further studies are needed to investigate the predictive value of LDL-C/HDL-C ratio in the progression of carotid AS and CAD over time.

Acknowledgments

We thank all of the participants for their cooperation in this study and the staff of the Xinjiang Key Laboratory of Cardiovascular Disease Research for their dedicated assistance in sample collection.

Contributions

Y-N Y, QZ and FL conceived and designed the study. Y-H W, QZ, H-M L, and J-Y L performed the experiments and collected the data. Y-N Y, Y-T M and X-M L completed the quality of data. FL and QZ performed the statistical analysis and wrote the paper. All authors read and approved the final manuscript.

Funding: This work was supported by the National Natural Science Foundation of China (No. 81460069, 81700315, U1503322), Science and Technology Agency of Xinjiang Uygur Autonomous Region (No. 2016E02072), and Postgraduate Scientific Research Project of Xinjiang Uygur Autonomous Region (No. XJGRI2017081).

Competing interests: None declared.

Ethics approval: The study was carried out in accordance with the declaration of Helsinki and the study protocol was approved by the Ethical Committee of First Affiliated Hospital of Xinjiang Medical University

Data sharing statement: No additional data are available.

Reference

For peer review only - http://bmjopen.bmj.com/site/about/guidelines.xhtml

1. Popkin BM. Is the obesity epidemic a national security issue around the globe? *Curr Opin Endocrinol Diabetes Obes* 2011;18(5):328-31. doi: 10.1097/MED.0b013e3283471c74

2. Wang Y, Mi J, Shan XY, et al. Is China facing an obesity epidemic and the consequences? The trends in obesity and chronic disease in China. *Int J Obes (Lond)* 2007;31(1):177-88. doi: 10.1038/sj.ijo.0803354

3. Guh DP, Zhang W, Bansback N, et al. The incidence of co-morbidities related to obesity and overweight: a systematic review and meta-analysis. *BMC Public Health* 2009;9:88. doi: 10.1186/1471-2458-9-88

4. Prospective Studies C, Whitlock G, Lewington S, et al. Body-mass index and cause-specific mortality in 900 000 adults: collaborative analyses of 57 prospective studies. *Lancet* 2009;373(9669):1083-96. doi: 10.1016/S0140-6736(09)60318-4

5. Balakumar P, Maung UK, Jagadeesh G. Prevalence and prevention of cardiovascular disease and diabetes mellitus. *Pharmacol Res* 2016;113(Pt A):600-09. doi: 10.1016/j.phrs.2016.09.040

6. Caleyachetty R, Thomas GN, Toulis KA, et al. Metabolically Healthy Obese and Incident Cardiovascular Disease Events Among 3.5 Million Men and Women. *J Am Coll Cardiol* 2017;70(12):1429-37. doi: 10.1016/j.jacc.2017.07.763

7. Lavie CJ, Milani RV. Obesity and cardiovascular disease: the hippocrates paradox? *J Am Coll Cardiol* 2003;42(4):677-9.

8. Lim S, Despres JP, Koh KK. Prevention of atherosclerosis in overweight/obese patients. - In need of novel multi-targeted approaches. *Circ J* 2011;75(5):1019-27.

9. Ceponiene I, Klumbiene J, Tamuleviciute-Prasciene E, et al. Associations between risk factors in childhood (12-13 years) and adulthood (48-49 years) and subclinical atherosclerosis: the Kaunas

For peer review only - http://bmjopen.bmj.com/site/about/guidelines.xhtml

Cardiovascular Risk Cohort Study. *BMC Cardiovasc Disord* 2015;15:89. doi: 10.1186/s12872-015-0087-0

10. Lee S, Cho GY, Kim HS, et al. Common carotid intima-media thickness as a risk factor for outcomes in Asian patients with acute ST-elevation myocardial infarction. *Can J Cardiol* 2014;30(12):1620-6. doi: 10.1016/j.cjca.2014.06.026

11. Simons PC, Algra A, Bots ML, et al. Common carotid intima-media thickness and arterial stiffness: indicators of cardiovascular risk in high-risk patients. The SMART Study (Second Manifestations of ARTerial disease). *Circulation* 1999;100(9):951-7.

12. Nambi V, Chambless L, He M, et al. Common carotid artery intima-media thickness is as good as carotid intima-media thickness of all carotid artery segments in improving prediction of coronary heart disease risk in the Atherosclerosis Risk in Communities (ARIC) study. *Eur Heart J* 2012;33(2):183-90. doi: 10.1093/eurheartj/ehr192

13. Bots ML, Hoes AW, Koudstaal PJ, et al. Common carotid intima-media thickness and risk of stroke and myocardial infarction: the Rotterdam Study. *Circulation* 1997;96(5):1432-7.

14. Prati P, Tosetto A, Vanuzzo D, et al. Carotid intima media thickness and plaques can predict the occurrence of ischemic cerebrovascular events. *Stroke* 2008;39(9):2470-6. doi: 10.1161/STROKEAHA.107.511584

15. Guo X, Li Z, Guo L, et al. An update on overweight and obesity in rural Northeast China: from lifestyle risk factors to cardiometabolic comorbidities. *BMC Public Health* 2014;14:1046. doi: 10.1186/1471-2458-14-1046

16. Sangros FJ, Torrecilla J, Giraldez-Garcia C, et al. Association of General and Abdominal Obesity With Hypertension, Dyslipidemia and Prediabetes in the PREDAPS Study. *Rev Esp Cardiol (Engl Ed)*

For peer review only

For peer review only - http://bmjopen.bmj.com/site/about/guidelines.xhtml

2017 doi: 10.1016/j.rec.2017.04.035

17. Skinner AC, Perrin EM, Moss LA, et al. Cardiometabolic Risks and Severity of Obesity in Children and Young Adults. *N Engl J Med* 2015;373(14):1307-17. doi: 10.1056/NEJMoa1502821

18. Cai L, Zhang L, Liu A, et al. Prevalence, awareness, treatment, and control of dyslipidemia among adults in Beijing, China. *J Atheroscler Thromb* 2012;19(2):159-68.

19. Nishikido T, Oyama J, Keida T, et al. High-dose statin therapy with rosuvastatin reduces small dense LDL and MDA-LDL: The Standard versus high-dose therApy with Rosuvastatin for lipiD lowering (SARD) trial. *J Cardiol* 2016;67(4):340-6. doi: 10.1016/j.jjcc.2015.05.017

20. Valensi P, Avignon A, Sultan A, et al. Atherogenic dyslipidemia and risk of silent coronary artery disease in asymptomatic patients with type 2 diabetes: a cross-sectional study. *Cardiovasc Diabetol* 2016;15(1):104. doi: 10.1186/s12933-016-0415-4

21. Enomoto M, Adachi H, Hirai Y, et al. LDL-C/HDL-C Ratio Predicts Carotid Intima-Media Thickness Progression Better Than HDL-C or LDL-C Alone. *J Lipids* 2011;2011:549137. doi: 10.1155/2011/549137

22. Katakami N, Kaneto H, Osonoi T, et al. Usefulness of lipoprotein ratios in assessing carotid atherosclerosis in Japanese type 2 diabetic patients. *Atherosclerosis* 2011;214(2):442-7. doi: 10.1016/j.atherosclerosis.2010.10.035

23. Ding D, Li X, Qiu J, et al. Serum lipids, apolipoproteins, and mortality among coronary artery disease patients. *Biomed Res Int* 2014;2014:709756. doi: 10.1155/2014/709756

24. Kunutsor SK, Zaccardi F, Karppi J, et al. Is High Serum LDL/HDL Cholesterol Ratio an Emerging Risk Factor for Sudden Cardiac Death? Findings from the KIHD Study. *J Atheroscler Thromb* 2017;24(6):600-08. doi: 10.5551/jat.37184

For peer review only

For peer review only - http://bmjopen.bmj.com/site/about/guidelines.xhtml

25. James PA, Oparil S, Carter BL, et al. 2014 evidence-based guideline for the management of high blood pressure in adults: report from the panel members appointed to the Eighth Joint National Committee (JNC 8). *JAMA* 2014;311(5):507-20. doi: 10.1001/jama.2013.284427

26. Polak JF, Pencina MJ, Pencina KM, et al. Carotid-wall intima-media thickness and cardiovascular events. *N Engl J Med* 2011;365(3):213-21. doi: 10.1056/NEJMoa1012592

27. National Cholesterol Education Program Expert Panel on Detection E, Treatment of High Blood Cholesterol in A. Third Report of the National Cholesterol Education Program (NCEP) Expert Panel on Detection, Evaluation, and Treatment of High Blood Cholesterol in Adults (Adult Treatment Panel III) final report. *Circulation* 2002;106(25):3143-421.

28. Fernandez-Friera L, Penalvo JL, Fernandez-Ortiz A, et al. Prevalence, Vascular Distribution, and Multiterritorial Extent of Subclinical Atherosclerosis in a Middle-Aged Cohort: The PESA (Progression of Early Subclinical Atherosclerosis) Study. *Circulation* 2015;131(24):2104-13. doi: 10.1161/CIRCULATIONAHA.114.014310

29. Buckley B. Letter to the editor regarding "Quantification of paraquat, MPTP, and MPP+ in brain tissue using microwave-assisted solvent extraction (MASE) and high-performance liquid chromatography-mass spectrometry". *Anal Bioanal Chem* 2013;405(1):439. doi: 10.1007/s00216-012-6503-8

30. Kim TJ, Shin HY, Chang Y, et al. Metabolically healthy obesity and the risk for subclinical atherosclerosis. *Atherosclerosis* 2017;262:191-97. doi: 10.1016/j.atherosclerosis.2017.03.035

31. Mancusi C, Gerdts E, Losi MA, et al. Differential effect of obesity on prevalence of cardiac and carotid target organ damage in hypertension (the Campania Salute Network). *Int J Cardiol* 2017;244:260-64. doi: 10.1016/j.ijcard.2017.06.045

For peer review only - http://bmjopen.bmj.com/site/about/guidelines.xhtml

32. Rosvall M, Persson M, Ostling G, et al. Risk factors for the progression of carotid intima-media thickness over a 16-year follow-up period: the Malmo Diet and Cancer Study. *Atherosclerosis* 2015;239(2):615-21. doi: 10.1016/j.atherosclerosis.2015.01.030

33. Sandfort V, Lai S, Ahlman MA, et al. Obesity Is Associated With Progression of Atherosclerosis During Statin Treatment. *J Am Heart Assoc* 2016;5(7) doi: 10.1161/JAHA.116.003621

34. Manninen V, Tenkanen L, Koskinen P, et al. Joint effects of serum triglyceride and LDL cholesterol and HDL cholesterol concentrations on coronary heart disease risk in the Helsinki Heart Study. Implications for treatment. *Circulation* 1992;85(1):37-45.

35. Hodis HN, Mack WJ, Azen SP, et al. Triglyceride- and cholesterol-rich lipoproteins have a differential effect on mild/moderate and severe lesion progression as assessed by quantitative coronary angiography in a controlled trial of lovastatin. *Circulation* 1994;90(1):42-9.

36. Nimkuntod P, Tongdee P. Plasma Low-Density Lipoprotein Cholesterol/High-Density Lipoprotein Cholesterol Concentration Ratio and Early Marker of Carotid Artery Atherosclerosis. *J Med Assoc Thai* 2015;98 Suppl 4:S58-63.

37. Tian L, Liu Y, Qin Y, et al. Association of the low-density lipoprotein cholesterol/high-density lipoprotein cholesterol ratio and concentrations of plasma lipids with high-density lipoprotein subclass distribution in the Chinese population. *Lipids Health Dis* 2010;9:69. doi: 10.1186/1476-511X-9-69

38. Chen QJ, Lai HM, Chen BD, et al. Appropriate LDL-C-to-HDL-C Ratio Cutoffs for Categorization of Cardiovascular Disease Risk Factors among Uygur Adults in Xinjiang, China. *Int J Environ Res Public Health* 2016;13(2):235. doi: 10.3390/ijerph13020235

39. Kurabayashi M, Yamazaki T, Group SS. Superior benefit of aggressive lipid-lowering therapy for high- risk patients using statins: the SUBARU study--more hypercholesterolemic patients achieve

For peer review only - http://bmjopen.bmj.com/site/about/guidelines.xhtml

Japan Atherosclerosis Society LDL-C goals with rosuvastatin therapy than with atorvastatin therapy. *J Atheroscler Thromb* 2008;15(6):314-23.

40. Jukema JW, Liem AH, Dunselman PH, et al. LDL-C/HDL-C ratio in subjects with cardiovascular disease and a low HDL-C: results of the RADAR (Rosuvastatin and Atorvastatin in different Dosages And Reverse cholesterol transport) study. *Curr Med Res Opin* 2005;21(11):1865-74. doi: 10.1185/030079905X74952

For peer review only

Fig 1. Comparison of LDL-C/HDL-C ratio between groups

The level of LDL-C/HDL-C ratio in CAP group was significantly higher than non-CAP group ($P < 0.001$).

Fig 2. ROC curve analysis of LDL-C/HDL-C ratio for predicting CCAP

The area under the curve (AUC) of LDL-C/HDL-C ratio was 0.748 (95 % CI: 0.720 - 0.776, $P < 0.001$). A ratio of 2.86 was identified as the optimal threshold with a sensitivity of 0.643 and specificity of 0.721.

For peer review only

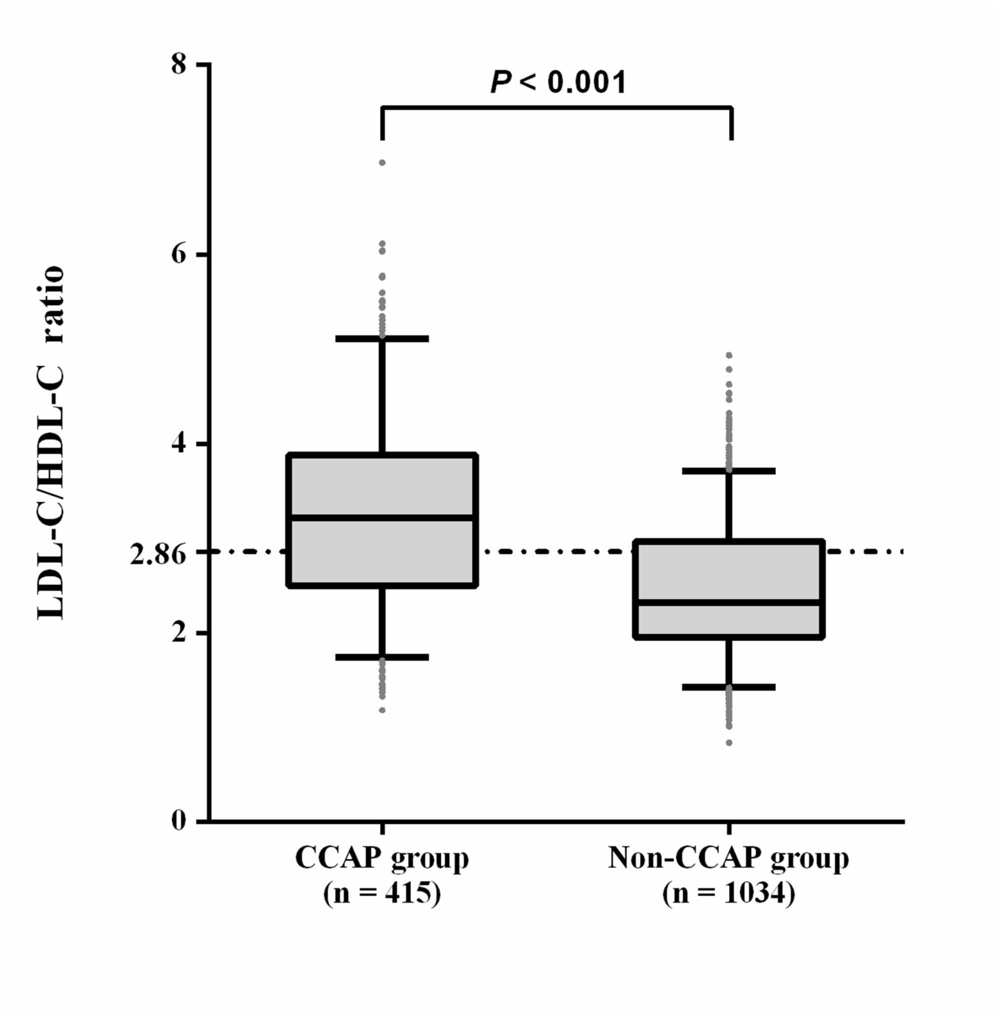

Fig 1. Comparison of LDL-C/HDL-C ratio between groups
The level of LDL-C/HDL-C ratio in CAP group was significantly higher than non-CAP group (P < 0.001).

111x112mm (300 x 300 DPI)

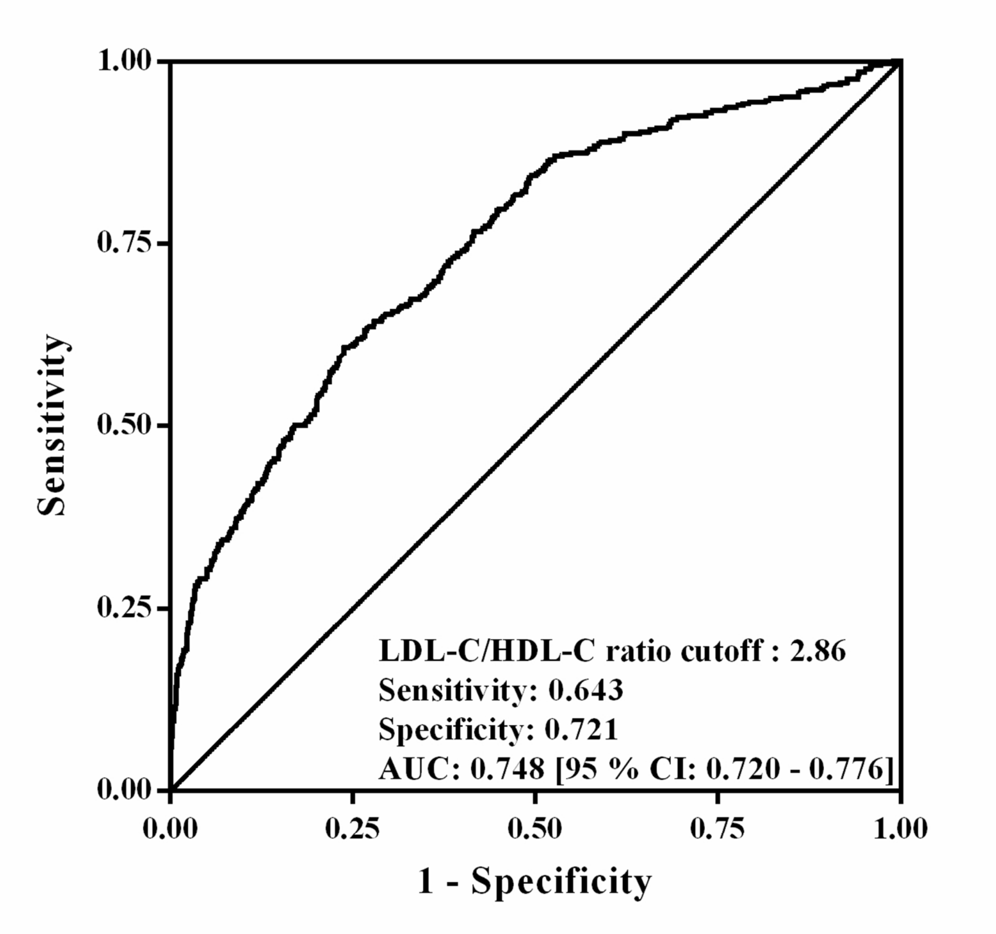

Fig 2. ROC curve analysis of LDL-C/HDL-C ratio for predicting CCAP
The area under the curve (AUC) of LDL-C/HDL-C ratio was 0.748 (95 % CI: 0.720 - 0.776, P < 0.001). A ratio of 2.86 was identified as the optimal threshold with a sensitivity of 0.643 and specificity of 0.721.

109x102mm (300 x 300 DPI)

STROBE Statement

Checklist of items that should be included in reports of observational studies

Section/Topic	Item No	Recommendation	Reported on Page No
**Title and abstract**	1	(*a*) Indicate the study's design with a commonly used term in the title or the abstract	2
		(*b*) Provide in the abstract an informative and balanced summary of what was done and what was found	2
**Introduction**			
Background/rationale	2	Explain the scientific background and rationale for the investigation being reported	4
Objectives	3	State specific objectives, including any prespecified hypotheses	5
**Methods**			
Study design	4	Present key elements of study design early in the paper	5
Setting	5	Describe the setting, locations, and relevant dates, including periods of recruitment, exposure, follow-up, and data collection	5
Participants	6	(*a*) *Cohort study*—Give the eligibility criteria, and the sources and methods of selection of participants. Describe methods of follow-up<br>*Case-control study*—Give the eligibility criteria, and the sources and methods of case ascertainment and control selection. Give the rationale for the choice of cases and controls<br>*Cross-sectional study*—Give the eligibility criteria, and the sources and methods of selection of participants	5-6
		(*b*) *Cohort study*—For matched studies, give matching criteria and number of exposed and unexposed<br>*Case-control study*—For matched studies, give matching criteria and the number of controls per case	
Variables	7	Clearly define all outcomes, exposures, predictors, potential confounders, and effect modifiers. Give diagnostic criteria, if applicable	5-6
Data sources/measurement	8*	For each variable of interest, give sources of data and details of methods of assessment (measurement). Describe comparability of assessment methods if there is more than one group	5-6
Bias	9	Describe any efforts to address potential sources of bias	5-6
Study size	10	Explain how the study size was arrived at	6
Quantitative variables	11	Explain how quantitative variables were handled in the analyses. If applicable, describe which groupings were chosen and why	7
Statistical methods	12	(*a*) Describe all statistical methods, including those used to control for confounding	7
		(*b*) Describe any methods used to examine subgroups and interactions	7
		(*c*) Explain how missing data were addressed	
		(*d*) *Cohort study*—If applicable, explain how loss to follow-up was addressed<br>*Case-control study*—If applicable, explain how matching of cases and controls was addressed<br>*Cross-sectional study*—If applicable, describe analytical methods taking account of sampling strategy	7
		(*e*) Describe any sensitivity analyses	7

For peer review only - http://bmjopen.bmj.com/site/about/guidelines.xhtml

Section/Topic	Item No	Recommendation	Reported on Page No
**Results**			
Participants	13*	(a) Report numbers of individuals at each stage of study—eg numbers potentially eligible, examined for eligibility, confirmed eligible, included in the study, completing follow-up, and analysed	7
		(b) Give reasons for non-participation at each stage	
		(c) Consider use of a flow diagram	
Descriptive data	14*	(a) Give characteristics of study participants (eg demographic, clinical, social) and information on exposures and potential confounders	7-8
		(b) Indicate number of participants with missing data for each variable of interest	
		(c) *Cohort study*—Summarise follow-up time (eg, average and total amount)	
Outcome data	15*	*Cohort study*—Report numbers of outcome events or summary measures over time	
		Case-control study—Report numbers in each exposure category, or summary measures of exposure	
		Cross-sectional study—Report numbers of outcome events or summary measures	7
Main results	16	(*a*) Give unadjusted estimates and, if applicable, confounder-adjusted estimates and their precision (eg, 95% confidence interval). Make clear which confounders were adjusted for and why they were included	8-10
		(*b*) Report category boundaries when continuous variables were categorized	11
		(*c*) If relevant, consider translating estimates of relative risk into absolute risk for a meaningful time period	
Other analyses	17	Report other analyses done—eg analyses of subgroups and interactions, and sensitivity analyses	11
**Discussion**			
Key results	18	Summarise key results with reference to study objectives	12
Limitations	19	Discuss limitations of the study, taking into account sources of potential bias or imprecision. Discuss both direction and magnitude of any potential bias	14-15
Interpretation	20	Give a cautious overall interpretation of results considering objectives, limitations, multiplicity of analyses, results from similar studies, and other relevant evidence	13-14
Generalisability	21	Discuss the generalisability (external validity) of the study results	15
**Other Information**			
Funding	22	Give the source of funding and the role of the funders for the present study and, if applicable, for the original study on which the present article is based	15

**Give information separately for cases and controls in case-control studies and, if applicable, for exposed and unexposed groups in cohort and cross-sectional studies.*

Note: An Explanation and Elaboration article discusses each checklist item and gives methodological background and published examples of transparent reporting. The STROBE checklist is best used in conjunction with this article (freely available on the Web sites of PLoS Medicine at http://www.plosmedicine.org/, Annals of Internal Medicine at http://www.annals.org/, and Epidemiology at http://www.epidem.com/). Information on the STROBE Initiative is available at www.strobe-statement.org.

For peer review only - http://bmjopen.bmj.com/site/about/guidelines.xhtml

LDL-C/HDL-C ratio and common carotid plaque in Xinjiang Uygur obese adults: a cross-sectional study

Journal:	*BMJ Open*
Manuscript ID	bmjopen-2018-022757.R1
Article Type:	Research
Date Submitted by the Author:	29-Jun-2018

Complete List of Authors:	Zhao, Qiang; First Affiliated Hospital of Xinjiang Medical University, Department of Cardiology<br>Liu, fen; First Affiliated Hospital of Xinjiang Medical University, Department of cardiology<br>Wang, Ying-Hong; First Affiliated Hospital of Xinjiang Medical University, Department of Health Management & Physical Examination Center<br>Lai, Hong-Mei; People's Hospital of Xinjiang Uygur Autonomous Region, Department of Cardiology<br>Zhao, Qian; First Affiliated Hospital of Xinjiang Medical University, Department of Cardiology<br>Luo, Jun-Yi; First Affiliated Hospital of Xinjiang Medical University, Department of Cardiology<br>Ma, Yi-Tong; First Affiliated Hospital of Xinjiang Medical University, Department of Cardiology<br>Li, Xiao-Mei; First Affiliated Hospital of Xinjiang Medical University, Department of Cardiology Urumqi, Xinjiang, CN<br>Yang, Yi-Ning; First Affiliated Hospital of Xinjiang Medical University, Department of Cardiology

**Primary Subject Heading**:	Cardiovascular medicine
Secondary Subject Heading:	Cardiovascular medicine
Keywords:	lipid ratio, carotid atherosclerotic plaque, obesity, Uygur

SCHOLARONE™ Manuscripts

LDL-C/HDL-C ratio and common carotid plaque in Xinjiang Uygur obese adults: a cross-sectional study

Qiang Zhao[1, 2, *], Fen Liu[1, 2, *], Ying-Hong Wang[1, 2, 3], Hong-Mei Lai[4], Qian Zhao[1, 2], Jun-Yi Luo[1, 2], Yi-Tong Ma[1, 2], Xiao-Mei Li[1, 2], Yi-Ning Yang[1, 2]

[1] Department of Cardiology, First Affiliated Hospital of Xinjiang Medical University, Urumqi, China;

[2] Xinjiang Key Laboratory of Cardiovascular Disease Research, Urumqi, China;

[3] Department of Health Management & Physical Examination Center, First Affiliated Hospital of Xinjiang Medical University, Urumqi, China;

[4] Department of Cardiology, People's Hospital of Xinjiang Uygur Autonomous Region, Urumqi, China.

* QZ and FL equally contributed.

Corresponding author: Yi-Ning Yang,
Department of Cardiology, First Affiliated Hospital of Xinjiang Medical University,
137 Liyushan Road, Urumqi, 830054 Xinjiang, China.
Tel: 86-991-4365381, e-mail: yangyn5126@163.com.

Short title: LDL-C/HDL-C ratio and common carotid artery plaque

Word count: 3440 Abstract: 238 Tables:4 Figures: 2

For peer review only - http://bmjopen.bmj.com/site/about/guidelines.xhtml

Abstract

Objective The aim of this study was to explore the relationship between LDL-C/HDL-C ratio and common carotid atherosclerotic plaque (CCAP) among obese adults of Uygur community in Xinjiang, China.

Design A hospital based cross-sectional study.

Setting First Affiliated Hospital of Xinjiang Medical University.

Participants A total of 1449 obese adults of Uygur population who were free of coronary artery disease were included in our study from 1 January 2014 to 31 December 2016.

Methodology Lipid profiles, other routine laboratory parameters and intima-media thickness of the common carotid artery were measured in all participants. Multivariate logistic regression analysis was used to examine the association between LDL-C/HDL-C ratio and CCAP.

Results Four hundred and fifteen (28.64 %) participants had CCAP. Participants with CCAP had significantly higher LDL-C/HDL-C ratio compared to those without CCAP (3.21[2.50, 3.88] vs. 2.33[1.95, 2.97], $P < 0.001$). Multivariate logistic regression analysis showed high LDL-C/HDL-C ratio as independent predictor of CCAP after adjusting for conventional cardiovascular risk factors. The top LDL-C/HDL-C ratio quartile (≥ 3.25) had an OR of 9.355 (95% CI: 6.181 - 14.157) compared with the bottom quartile (< 2.07) of LDL-C/HDL-C ratio. ($P < 0.001$) after adjustment for age, BMI, smoking and diabetes mullites.

Conclusion CCAP is highly prevalent in Uygur obese adults. A high LDL-C/HDL-C ratio is an independent predictor of CCAP. It may help identify obese individuals who are at high risk of CCAP and who may benefit from intensive LDL-lowering therapy.

Keywords: lipid ratio; carotid atherosclerotic plaque; obesity; Uygur

For peer review only

For peer review only - http://bmjopen.bmj.com/site/about/guidelines.xhtml

Strengths and limitations of this study

1.The survey sample was demographically representative of Uygur obese adults aged 18 - 80 years in Xinjiang.

2.This is the first study to date that investigate the association of LDL-C/HDL-C ratio and common carotid plaque in Uygur obese adults.

3.The is a cross-sectional study, we cannot show causality between common carotid plaque and risk factors.

4.The cross-sectional design did not allow us to examine the effects of LDL/HDL-C ratio on the progression of carotid plaque.

For peer review only

For peer review only - http://bmjopen.bmj.com/site/about/guidelines.xhtml

1. Introduction

Prevalence of obesity has been increasing in China during the past few decades. Presently, overweight or obese people account for one third of Chinese adults. [1 2] Cardiometabolic risk factors including hypertension, dyslipidemia, and type 2 diabetes mellitus (T2DM) are more prevalent in obese adults, partly explaining the association between obesity and increased cardiovascular diseases (CVD). [3-5] Recent data demonstrated that obese individuals who were even metabolically healthy had a higher risk of CVD including coronary artery disease (CAD), heart failure and cerebrovascular disease compared to the normal weight metabolically healthy individuals. [6] Obesity has been identified as an independent risk factor for CVD and ultimately death, [3 7] and it is emerging as a major public health challenge in China.

Atherosclerosis (AS) is regarded as the primary cause of mortality and morbidity in CVD. [8] It is a well-known fact that AS is a systemic process beginning at early childhood and typically remains asymptomatic until later in life. [9] Approximately 30% of first acute cardiovascular events are fatal, hence the prevention of CVD ought to be directed towards the risk factors including the silent preclinical phase of the process. Using a non-invasive ultrasound biomarker of AS, the carotid intima-media thickness (CIMT) has been demonstrated as a reliable substitute of determining risk of cardiac events in asymptomatic adults. [10 11] Of all the segments of the carotid artery used in CIMT, the common carotid artery (CCA) is the easiest to image and got the highest reproducibility. Data from the Atherosclerosis Risk in Communities (ARIC) study showed that CCA-CIMT detection was more reliable than measuring CIMT of all carotid artery segments, providing a good marker for CAD risk prediction. [12] Prior researchers have described that the presence of common carotid atherosclerotic plaque (CCAP) was correlated with the increased prevalence of CAD, myocardial infarction, stroke and CVD mortality beyond traditional cardiovascular risk factors. [11-15] Thus, early detection and initiation of treatment of obese patients with CCAP might improve the prevention of cardiovascular events.

Many cohort and nested case-control studies showed that obesity is closely related with high prevalence of dyslipidemia, characterized by increased plasma triglycerides (TG), total cholesterol (TC), and low-density lipoprotein cholesterol (LDL-C), together with lower

For peer review only - http://bmjopen.bmj.com/site/about/guidelines.xhtml

high-density lipoprotein cholesterol (HDL-C) levels. [16-19] Previous studies have established the important role of LDL-C in the pathogenesis of AS. LDL-C has been used to monitor the lipid-lowering response to treatments in almost all patients with CVD. However, a number of clinical trials showed 60 - 70 % of CVD events continue to occur despite LDL-lowering therapy, which may be due to residual risks associated with lipid abnormalities especially atherogenic dyslipidemia, including increased TG levels and reduced HDL-C levels. [20 21] Therefore, a need for new targets to compliment the measures of LDL-C lowering has been suggested. Recent studies identified that LDL-C/HDL-C ratio as a better biomarker to predict both AS progression and CVD than LDL-C or HDL-C alone. [22-25] However, data on the associations between LDL-C/HDL-C ratio and CCAP among obese population is limited. Thus, the present study was designed to explore the relationship between the level of LDL-C/HDL-C ratio and CCAP among Uygur obese adults in Xinjiang to prove this fact.

2. Subjects and methods

Study design and participants

This is a cross-sectional study conducted at the First Affiliated Hospital of Xinjiang Medical University from 1 January 2014 to 31 December 2016 in Xinjiang, China.

The study population was signed-up from among the outpatients and inpatients admitted to the cardiology department and participants in routine health examinations at the health physical examination center of the First Affiliated Hospital of Xinjiang Medical University. Eligible participants were obese Uygur adults aged 18 - 80 years who were free of clinically evident CAD, which was defined as absence of any previous myocardial infarction, previous or current angina with objective evidence of atherosclerotic CAD, or a previous coronary revascularization procedure. The major exclusion criteria included history of physician-diagnosed CAD and stroke, stenosis of carotid artery $>$30 % on angiography, heart failure, congenital heart disease, current cancer, chronic kidney or liver diseases, and thyroid diseases.

Ethical statement

The Ethics Committee of the Faculty of Medicine at First Affiliated Hospital of Xinjiang Medical University approved this study. All participants provided written informed consent.

For peer review only - http://bmjopen.bmj.com/site/about/guidelines.xhtml

Risk factors and definitions

Thorough medical history was obtained and a routine clinical examination including physical examination, blood biochemical examination and carotid ultrasonography was undertaken in all participants. Data on age, gender, cigarette smoking status, body mass index (BMI), systolic blood pressure, diastolic blood pressure, past medical history including hypertension, diabetes mullites (DM), previous medication, creatinine level, uric acid, high-sensitivity C-reactive protein (hs-CRP), and lipid profiles were recorded.

Hypertension was defined as having a history of hypertension and/or an average systolic blood pressure ≥ 140 mmHg and/or an average diastolic blood pressure ≥ 90 mmHg or if participants were taking antihypertensive medication. [26] The presence of DM was based on a history of diabetes-defined as either a current or previous fasting plasma glucose level of 7 mmol/L or higher or when participants were taking antidiabetic medication. [27] BMI was calculated by dividing the weight in kilograms by the height in meters square. Obesity was defined as BMI ≥ 28 kg/m^2 by Chinese standards. [2] Smoking was labeled if participant was currently smoking or had smoked in the previous 6 months.

Lipid profile analyses

Overnight fasting venous blood samples were collected from all subjects for the assessment of routine laboratory parameters. Laboratory tests were performed at the core laboratory of the First Affiliated Hospital of Xinjiang Medical University. Fasting plasma glucose, blood urea nitrogen, creatinine, uric acid, and hs-CRP were determined by the standard methods. Triglycerides (TG), total cholesterol (TC), LDL-C and HDL-C levels were directly measured using the homogeneous enzymatic colorimetric assay (Roche Diagnostics) on an automatic analyzer (Cobas 8000). Dyslipidemia was calculated on the basis of having at least one of the following conditions: TC concentration > 6.22 mmol/L, LDL-C concentration > 4.14 mmol/L, HDL-C concentration < 1.04 mmol/L, TG concentration > 2.26 mmol/L, or a history of use of lipid-lowering drugs. [28]

Carotid ultrasonography assessments

The Acuson Sequoia 512 instrument ultrasound mainframes with a 7.5-Mhz linear array transducer (Siemens AG, Erlangen, Germany) was used to acquire ultrasonographic images of

For peer review only - http://bmjopen.bmj.com/site/about/guidelines.xhtml

both the right and left CCAs. Intima–media interface lines were manually traced as continuous lines by certified sonographers. The IMT was measured over such a segment of the CCA that was 1 cm long, located approximately 0.5 cm below the carotid-artery bulb, and considered not to contain any plaque. It was measured as the distance between the media-adventitia and intima-lumen interfaces. [27] We performed three views on each side of CCAs to determine the maximal and mean CIMT. The presence of CCAP was defined as a CIMT > 1.5 mm, or a focal structure encroaching into the arterial lumen of at least 0.5 mm, or 50 % of the surrounding CIMT value.[29] Carotid ultrasonography was performed and evaluated by two independent ultrasonologists who were unaware of the clinical and laboratory data of any of the participants. A third sinologist was consulted if there was a discrepancy between the diagnostic results of the two ultrasonologists. Consensus was then accomplished in all patients.

Statistical analysis

Statistical analyses were conducted using SPSS version 17.0 (SPSS Inc., Chicago, Illinois). Normally distributed continuous data were presented as mean ± standard deviation and compared using unpaired t-tests, while non-normally distributed data were summarized as median with interquartile range (IQR) and compared using Mann-Whitney or Kruskal-Wallis tests. Categorical variables were summarized in numbers and percentages and compared between groups with Chi-square tests. Subjects were also classified according to LDL-C/HDL-C ratio quartiles, and the clinical characteristics were analyzed using Mann-Whitney tests or Chi-square tests. For subsequent pairwise comparisons, a 2-sided Bonferroni P value of 0.05 divided by 6 (0.0083) was used to establish statistical significance. LDL-C/HDL-C ratio was included in the model as a quartile categorical variable. Univariate logistic regression model was used to estimate the independent predictors of CCAP. After performing univariate analysis, significant variables (age, BMI, smoking, DM, TC, and LDL-C/HDL-C ratio quartiles) were used in multivariate logistic regression analysis. Area under the ROC curve (AUC) and 95% confidence intervals (CI) was calculated. A 2-tailed value of $P < 0.05$ was considered significant.

For peer review only - http://bmjopen.bmj.com/site/about/guidelines.xhtml

Patient and public involvement

The study was designed to investigate the association of lipids ratio and common carotid artery plaque. However, no patients or members of the public were included in the design, recruitment or conduct of the study. The results of measurements would be disseminated to participants after the study which was completed by the study team. The burden of the intervention will not be assessed by patients themselves.

3. Results

A total of 1449 participants with a mean age of 53.81 ± 11.52 years having no history of CAD were recruited in this study. All participants had grouped according to the presence of CCAP. There were 415 (28.64 %) participants in the CCAP group (mean age 55.29 ± 11.04 years and 53.98 % were men) and 1034 participants (71.36 %) in the non-CCAP group (mean age 53.22 ± 11.67 years and 51.93 % were men).

Baseline characteristics of the study population

The baseline characteristics of all participants according to CCAP status were shown in Table 1. There was no significant difference between CCAP group and non-CCAP group with respect to gender, systolic blood pressure, diastolic blood pressure, prevalence of hypertension, and previous medications. The participants in the CCAP group were more likely to be older with higher BMI and had higher prevalence of diabetes and smoking than those in the non-CCAP group (All $P < 0.05$).

Table 1. Baseline characteristics of study population according to CCAP status.

Characteristics	Overall (n = 1449)	CCAP group (n = 415)	Non-CCAP group (n = 1034)	*P*-value
Demographics				
Gender				0.482
Women, n (%)	688 (47.48)	191 (46.02)	497 (48.07)	
men, n (%)	761 (52.52)	224 (53.98)	537 (51.93)	
Age, years	53.81 ± 11.52	55.29 ± 11.04	53.22 ± 11.67	0.002
Vital signs				

For peer review only - http://bmjopen.bmj.com/site/about/guidelines.xhtml

Body mass index, kg/m^2	30.12 (28.90, 32.32)	30.39 (29.06, 32.74)	30.10 (28.76, 32.01)	0.027
Systolic blood pressure, mmHg	130.19 ± 14.13	130.99 ± 12.94	129.88 ± 14.58	0.176
Diastolic blood pressure, mmHg	80.98 ± 8.42	80.67 ± 8.10	81.11 ± 8.54	0.369
Medical histories				
Smoking, n (%)	246 (16.98)	90 (21.69)	156 (15.09)	0.002
Hypertension, n (%)	444 (30.64)	123 (29.64)	321 (31.04)	0.600
DM, n (%)	324 (22.36)	133 (32.05)	191 (18.47)	< 0.001
Previous Medications				
Antihypertensive drugs, n (%)	311 (21.46)	83 (20.00)	228 (22.05)	0.390
Antidiabetic agents, n (%)	147 (10.14)	51 (12.29)	96 (9.28)	0.087
Statins, n (%)	146 (10.08)	37 (8.92)	109 (10.54)	0.353

CCAP, common carotid atherosclerotic plaque.

Laboratory and Carotid ultrasonography parameters of study population

Laboratory and carotid ultrasonography parameters according to CCAP status were summarized in Table 2. Out of 1449 participants, 48.17 % of them had dyslipidemia. No significant differences were observed in the serum levels of TG, hs-CRP, blood urea nitrogen, creatinine, uric acid and transaminases between CCAP and non-CCAP group. Participants with CCAP had significantly higher concentrations of fasting plasma glucose, TC, LDL-C, and higher prevalence of dyslipidemia as well as lower HDL-C level than those in the non-CCAP group. Furthermore, the LDL-C/HDL-C ratio was significantly higher in the CCAP group (3.21[2.50, 3.88] vs. 2.33[1.95, 2.97], $P < 0.001$) (Fig 1).

Table 2. Laboratory and carotid ultrasonography parameters of study population according to CCAP status

Characteristics	Overall (n = 1449)	CCAP group (n = 415)	Non-CCAP group (n = 1034)	*P*-value
Laboratory measurements				
White blood cell count, 10^9/L	6.82 ± 1.87	6.71 ± 1.92	6.87 ± 1.86	0.147
Platelet count, 10^9/L	212.13 ± 50.79	213.45 ± 51.19	211.60 ± 50.65	0.531
Hemoglobin, g/L	137.02 ± 15.71	136.49 ± 15.42	137.24 ± 15.82	0.408

For peer review only - http://bmjopen.bmj.com/site/about/guidelines.xhtml

Fasting plasma glucose, mmol/L	5.28 (4.66, 5.93)	5.73 (5.17, 6.48)	5.08 (4.59, 5.65)	< 0.001
TG, mmol/L	1.60 (1.11, 2.15)	1.53 (1.14, 2.00)	1.62 (1.10, 2.16)	0.375
TC, mmol/L	4.89 ± 0.87	5.11 ± 0.84	4.80 ± 0.87	< 0.001
LDL-C, mmol/L	3.04 ± 0.66	3.44 ± 0.69	2.88 ± 0.58	< 0.001
HDL-C, mmol/L	1.16 (1.00, 1.35)	1.10 (0.92, 1.28)	1.19 (1.03, 1.38)	< 0.001
LDL-C/HDL-C ratio	2.52 (2.07, 3.25)	3.21 (2.50, 3.88)	2.33 (1.95, 2.97)	< 0.001
Dyslipidemia, n (%)	698 (48.17)	267 (64.34)	431 (41.68)	< 0.001
Hs-CRP, mg/L	2.66 (1.27, 4.42)	2.71 (1.33, 4.35)	2.64 (1.25, 4.44)	0.734
Blood urea nitrogen, mmol/L	4.87 (4.10, 5.80)	4.97 (4.10, 5.90)	4.80 (4.10, 5.71)	0.284
Creatinine, μmol/L	70.90 ± 15.34	71.82 ± 15.68	70.54 ± 15.19	0.150
Uric acid, mg/dL	5.16 ± 1.40	5.23 ± 1.43	5.13 ± 1.39	0.214
Glutamic-pyruvic transaminase, U/L	27.56 ± 15.64	28.44 ± 16.15	27.21 ± 15.42	0.179
Glutamic-oxaloacetic transaminase, U/L	19.80 (16.50, 24.21)	19.60 (16.60, 24.20)	19.80 (16.50, 24.23)	0.850
Ultrasonographic parameters				
Maximal CCA-IMT, mm	1.10 (0.80, 1.40)	1.60 (1.50, 1.80)	0.90 (0.70, 1.10)	< 0.001
Mean CCA-IMT, mm	0.80 (0.60, 1.10)	1.30 (1.10, 1.40)	0.80 (0.60, 0.90)	< 0.001
Unilateral plaque, n (%)	227 (15.67)	227 (54.70)	-	-
Bilateral plaque, n (%)	188 (12.97)	188 (45.30)	-	-
Maximal CCA-IMT > 0.9 mm, n (%)	429 (29.61)	-	429 (41.49)	-

TG, triglyceride; TC, total cholesterol; LDL-C, low density lipoprotein-cholesterol; HDL-C, high density lipoprotein-cholesterol; CCA-IMT, common carotid artery intima–media thickness.

Out of 415 participants in the CCAP group, 54.70 % participants had unilateral and 45.30 % participants had bilateral plaques. The maximal and mean CCA-IMT were 1.60 mm (IQR: 1.50 - 1.80) and 1.30 mm (IQR: 1.10 - 1.40) in the CCAP group, respectively. In

For peer review only - http://bmjopen.bmj.com/site/about/guidelines.xhtml

non-CCAP group, 41.49 % participants had maximum CCA-IMT higher than 0.90 mm, which represented carotid intimal thickening, and the maximal and mean CCA-IMT were 0.90 mm (IQR: 0.70 - 1.10) and 0.80 mm (IQR: 0.60 - 0.90), respectively. Participants in the CCAP group had higher maximal and mean CCA-IMT than those in non-CCAP group (All $P <$ 0.001).

As shown in Table 3, participants' baseline characteristics were presented by LDL-C/HDL-C ratio quartiles. There were no significant differences in gender, age, prevalence of hypertension, and use of drugs between each quartile. Compared with participants in the first quartile of LDL-C/HDL-C ratio, those in the fourth quartile were more frequently smokers, and had higher prevalence of DM and dyslipidemia. They also had higher prevalence of CCAP and maximal and mean CCA-IMT.

Table 3. Clinical characteristics of study population stratified by LDL-C/HDL-C ratio quartiles

Characteristics	LDL-C/HDL-C Ratio Quartiles				*P*-value
	First (< 2.07) n = 362	Second (≥ 2.07 - <2.52) n = 362	Third (≥ 2.52 - <3.25) n = 362	Fourth (≥ 3.25) n = 363	
Gender					0.160
Women, n (%)	189 (52.21)	161 (44.48)	173 (47.79)	165 (45.45)	
Men, n (%)	173 (47.79)	201 (55.52)	189 (52.21)	198 (54.55)	
Age, years	52.00 (43.00, 64.00)	54.00 (45.00, 62.00)	52.00 (45.00, 63.00)	53.00 (46.00, 63.00)	0.370
Body mass index, kg/m^2	30.21 (29.04, 32.38)	29.74 (28.67, 31.25)	30.11 (28.99, 32.45) Ω	30.49 (29.05, 32.69) †	< 0.001
Smoking, n (%)	42 (11.60)	62 (17.13)	58 (16.02)	84 (23.14) &	0.001
Hypertension, n (%)	118 (32.60)	102 (28.18)	109 (30.11)	115 (31.68)	0.590
Antihypertensive drugs, n (%)	83 (22.93)	69 (19.06)	80 (22.10)	79 (21.76)	0.613
Diabetes mellitus, n (%)	53 (14.64)	60 (16.57)	88 (24.31) # Ω	123 (33.88) & † ‡	< 0.001
Antidiabetic agents, n (%)	25 (6.91)	35 (9.67)	41 (11.33)	46 (12.67)	0.061
Dyslipidemia, n (%)	79 (21.82)	113 (31.22) *	180 (49.72) # Ω	326 (89.81) & † ‡	< 0.001
Statins, n (%)	31 (8.56)	42 (11.60)	40 (11.05)	33 (9.09)	0.455

For peer review only - http://bmjopen.bmj.com/site/about/guidelines.xhtml

For peer review only

CCAP, n (%)	35 (9.67)	72 (19.89) *	108 (29.83) # Ω	200 (55.10) & † ‡	< 0.001
Maximal CCA-IMT, mm	1.00 (0.80, 1.20)	1.00 (0.80, 1.30)	1.00 (0.80, 1.40)	1.30 (0.90, 1.60) & † ‡	< 0.001
Mean CCA-IMT, mm	0.80 (0.60, 1.00)	0.80 (0.60, 1.00)	0.80 (0.60, 1.10)	1.00 (0.70, 1.30) & † ‡	< 0.001

Date were compared by Mann-Whitney tests or Chi-square tests. *P <0.0083 when comparing the first quartile vs. the second quartile. #P <0.0083 when comparing the first quartile vs. the third quartile. &P <0.0083 when comparing the first quartile vs. the fourth quartile. ΩP <0.0083 when comparing the second quartile vs. the third quartile. †P <0.0083 when comparing the second quartile vs. the fourth quartile. ‡P <0.0083 when comparing the third quartile vs. the fourth quartile.

Association of LDL-C/HDL-C ratio and CCAP

Univariate and multivariate logistic regression models of the associations between LDL-C/HDL-C ratio and CCAP were described in Table 4. To determine the linear trends of the risk, we explored the ORs of CCAP by LDL-C/HDL-C ratio quartile groups, with the first quartile serving as the reference category. Entering LDL-C/HDL-C ratio as quartiles to the CCAP univariable model revealed that the top quartile (fourth quartile, ≥ 3.25) had an OR of 11.464 (95% CI: 7.644 - 17.193) compared with the bottom quartile (first quartile, < 2.07) (P < 0.001). LDL-C (OR: 3.012, 95 % CI: 2.274 - 3.917, P < 0.001) and HDL-C (OR: 0.222, 95 % CI: 0.415 - 0.340, P < 0.001) were also found to be independent predictors of CCAP in univariate analysis. Other significant correlates of CCAP were age (> 45 years, every 10 years), BMI, smoking, DM, and TC (all P < 0.05). Because LDL-C/HDL-C ratio integrates both LDL-C and HDL-C, and is strongly correlated with the LDL-C and HDL-C, only LDL-C/HDL-C ratio was involved into multivariate analysis.

After adjusting for age, BMI, smoking, presence of DM, and mean TC level, the association between LDL-C/HDL-C ratio and CCAP still remained significant (OR [95% CI]: second quartile, 2.184 [1.404 - 3.399]; third quartile, 3.556 [2.333 - 5.421]; top quartile, 9.355 [6.181 - 14.157]). The multivariable-adjusted ORs of CCAP increased continuously and linearly, and statistical significance was observed from the second quartile of LDL-C/HDL-C ratio. The top LDL-C/HDL-C ratio quartile had an OR of 9.355 (95% CI: 6.181 - 14.157) compared with the bottom quartile (P < 0.001) after adjustment for age, BMI, smoking, DM and TC.

For peer review only

For peer review only - http://bmjopen.bmj.com/site/about/guidelines.xhtml

Table 4. Logistic regression analysis of the risk factors for CCAP

Variables	Univariate			Multivariate		
	OR	95 % CI	*P*-value	OR	95 % CI	*P*-value
Gender				-		
Female	Reference			-		
Male	1.085	0.864 - 1.364	0.482	-		
Age			0.013	-		0.040
<45 years	Reference			Reference		
45 - 54 years	1.631	1.178 - 2.257	0.003	1.425	0.999 - 2.034	0.051
55 - 64 years	1.485	1.048 - 2.104	0.026	1.584	1.081 - 2.322	0.018
65 - 74 years	1.625	1.132 - 2.333	0.009	1.642	1.101 - 2.451	0.015
≥ 75 years	2.288	1.220 - 4.292	0.010	2.375	1.176 - 4.799	0.016
Body mass index	1.068	1.024 - 1.114	0.002	1.058	1.010 - 1.108	0.018
Smoking	1.559	1.167 - 2.081	0.003	1.407	1.019 - 1.943	0.038
Hypertension	0.936	0.730 - 1.200	0.600	-		
Diabetes mellitus	2.082	1.606 - 2.697	< 0.001	1.590	1.196 - 2.113	0.001
TG	0.935	0.817 - 1.070	0.327	-		
TC	1.512	1.323 - 1.728	< 0.001	1.437	1.244 - 1.659	< 0.001
LDL-C	3.012	2.274 - 3.917	< 0.001	-		
HDL-C	0.222	0.145 - 0.340	< 0.001	-		
LDL-C/HDL-C ratio			< 0.001			< 0.001
First quartile (< 2.07)	Reference			Reference		
Second quartile (≥2.07 - <2.52)	2.320	1.503 - 3.579	< 0.001	2.184	1.404 - 3.399	0.001
Third quartile (≥2.52 - <3.25)	3.973	2.623 - 6.016	< 0.001	3.556	2.333 - 5.421	< 0.001
Fourth quartile (≥ 3.25)	11.464	7.644 - 17.193	< 0.001	9.355	6.181 - 14.157	< 0.001
High sensitivity C-reactive protein	1.006	0.966 - 1.049	0.760	-		
Uric acid	1.053	0.971 - 1.141	0.214	-		
Antihypertensive drugs	0.884	0.667 - 1.172	0.390	-		
Antidiabetic agents	1.369	0.955 - 1.963	0.088	-		
Statins	0.831	0.561 - 1.229	0.353	-		

The ROC curve of LDL-C/HDL-C ratio for predicting CCAP was listed in Fig. 2. The AUC with LDL-C/HDL-C ratio used to detect CCAP was 0.748 (95 % CI: 0.720 - 0.776, P <

For peer review only

For peer review only - http://bmjopen.bmj.com/site/about/guidelines.xhtml

0.001).

4. Discussion

In the present study, we examined the associations between LDL-C/HDL-C ratio and CCAP in Uygur obese adults. The main findings of our study are as follows: CCAP is highly prevalent in Uygur obese adults. We observed remarkable differences in the risk of CCAP among subgroups as defined by LDL-C/HDL-C ratio levels, the lowest rate of CCAP (9.67%) was observed in the lowest LDL-C/HDL-C ratio (First quartile) subgroup, whereas the highest rate (55.10%) was observed in the highest LDL -C/HDL-C ratio (Fourth quartile) subgroup. We found that LDL-C/HDL-C ratio is an independent predictor of CCAP after adjusting for possible confounding factors including mean TC level. There was a positive association between the increase in LDL-C/HDL-C ratio and the prevalence of CCAP. These findings, if confirmed, would help identify those obese individuals who are at high risk of CCAP.

Epidemiologic studies have identified obesity as a risk factor for an expanding set of chronic diseases, including CVD, DM, dyslipidemia, hypertension, and chronic kidney disease.[30 31] Recent studies have reported that obesity increased the risk of subclinical carotid AS. [32-34] In this study, 28.64 % obese participants had CCAP. The best predictive marker of CCAP was high LDL-C/HDL-C ratio, and the serum LDL-C and HDL-C also discriminated CCAP. The demonstrated high prevalence of CCAP in obese individuals expands the findings made in the previous population-based studies in which the association of obesity with atherosclerotic progression has been proven. [33 35 36]

The causal relationship between LDL-C and increased risk of CAD has been well established. However, even aggressive reduction in LDL-C using statin agents still leaves considerable residual risk of CAD events. Early epidemiological studies demonstrated lower HDL-C level is related with an increased risk of CAD. However, some recent studies failed to show significant association of HDL-C levels and outcomes for patients with CAD on intensive lipid therapy or patients with CAD undergoing elective coronary artery bypass grafting. Nowadays, the focus of the HDL-C hypothesis has been directed toward the function

For peer review only - http://bmjopen.bmj.com/site/about/guidelines.xhtml

of HDL-C.

The LDL-C/HDL-C ratio has recently been recognized as a new biometric that has clinical relevance in AS diseases. In the Helsinki Heart Study analyses, LDL-C/HDL-C ratio was shown to be the best single predictor of cardiac events. [37] Kunutsor SK et al [25] conducted a study investigating the association between LDL-C/HDL-C ratio and sudden cadiac death (SCD). High LDL-C/HDL-C ratio was found to be independently associated with an increased risk of SCD. There was approximately a two-fold increase in the risk of SCD comparing the top versus bottom quintile of serum LDL-C/HDL-C ratio. Another study reported elevated LDL-C/HDL-C ratio was related with the progression of coronary artery lesions. [25 38]

Recently, several investigators have examined the utility of LDL-C/HDL-C ratio in predicting carotid AS. However, contrasting results have been demonstrated. Enomoto M et al [22] conducted a periodic epidemiological survey to investigate the relationship between lipid profiles and the progression of common carotid IMT. They found LDL-C/HDL-C ratio was a better marker in predicting IMT progression than HDL-C or LDL-C alone during an 8-year follow up survey. Katakami N et al [23] explored the relationships between various lipid parameters including lipid ratios and carotid AS in 934 T2DM patients having no obvious atherosclerotic diseases in Japan. They concluded that LDL-C/HDL-C ratio was positively associated with carotid atherosclerotic plaques. Wu TW et al [39] surveyed the impact of LDL-C/HDL-C ratio on CIMT among 1579 residents aged 40 - 74 years in northern Taiwan. The results demonstrated LDL-C/HDL-C ratio was strongly correlated with CIMT in both males and females, indicating that LDL-C/HDL-C ratio was an important determinant of increased CIMT.

However, Nimkuntod P et al [40] failed to identify any association between LDL-C/HDL-C ratio and early carotid atherosclerosis CIMT carotid plaques as well as carotid plaque type. The small sample of the study may possibly explain the discrepancy. In our study, we found obese individuals with CCAP had significantly higher prevalence of dyslipidemia. Several pathophysiological processes have been proposed to explain the mechanisms of dyslipidemia in obese population. First, dyslipidemia was mainly driven by the increased lipid production

For peer review only - http://bmjopen.bmj.com/site/about/guidelines.xhtml

and hydrolysis of TG. Second, higher plasma level of free fatty acid may also contribute to dyslipidemia. According to our findings, high LDL-C/HDL-C ratio is the strongest predictors of CCAP after adjusting for major risk factors, thus indicating that the high LDL-C/HDL-C ratio may influence the progression of CCAP through pathways beyond traditional risk factors.

Our finding is consistent with previous studies, and revealed a positive association between high LDL-C/HDL-C ratio and CCAP in Uygur obese adults. While the exact mechanism of the superiority of LDL-C/HDL-C ratio to either individual LDL-C or HDL-C remains to be elucidated, several explanations could be suggested. First, LDL-C/HDL-C ratio represents the proportion between the atherogenic and anti-atherosclerotic lipoproteins, which has greater predictive value for atherosclerotic changes. Second, LDL-C/HDL-C ratio was closely correlated with the distribution of HDL subclasses. With an increase in the LDL-C/HDL-C ratio, there was a general shift toward smaller-sized HDL particles, implying that the maturation process of HDL was blocked, which may contribute to the progression of AS. [41] Third, high LDL-C/HDL-C ratio may lead to the fluctuations in the composition of atherosclerotic plaques. High LDL-C/HDL-C ratio may be associated with the development of large lipid-rich necrotic core.

Our previous work described the cutoff value of LDL-C/HDL-C ratio for assessing CVD risk factors was 2.5 among Uygur adults. [42] In the present study, participants with LDL-C/HDL-C ratio ≥ 2.07 had a significantly higher risk of CCAP, which was higher than that of obtained by Kurabayashi M et al. [43] They found the cutoff value of LDL-C/HDL-C ratio for initiating secondary prevention of AS was > 1.5, and the cutoff ratio for initiating primary prevention of AS was > 2.0. The discordance between our results may be due to different characteristics between our population and theirs. The participants recruited into our study were obese individuals, and the mean level of LDL-C is significantly higher than those previous studies, which may help to explain the high LDL-C/HDL-C ratio in our study. Secondly, the distinct ethnic origin, diet, life styles, living habits and living environments that are specific to Uygur population may contribute to these discrepancies. Further studies are needed to confirm our finding of high LDL-C/HDL-C ratio

For peer review only - http://bmjopen.bmj.com/site/about/guidelines.xhtml

Several limitations of this study should be considered. First, our findings are based on a population from a single center, and should be verified in other larger populations. Second, this is a cross-sectional analysis, which limits the temporal interpretation of predictive measurements. Third, we only measured baseline LDL-C/HDL-C ratio, and the relations between LDL-C/HDL-C ratio changes and outcomes could not be ascertained. In future studies, LDL-C/HDL-C ratio during the follow-up period should also be examined. Fourth, the absence of a health control group should be considered as the main constraint of our study. Whether the results can be applied to general obese population remains to be investigated. Finally, statins are known to affect changes in LDL-C/HDL-C ratio. [44] About 10% participants had taken statin medication in our study, so we can't evaluate its influence on lipid ratio changes and the progression of CCAP.

In summary, our study demonstrated that high LDL-C/HDL-C ratio is independently associated with increased risk of CCAP among Uygur obese adults in Xinjiang, China. LDL-C/HDL-C ratio may help identify obese individuals who are at high risk of CCAP and who may benefit from intensive LDL-lowering therapy. Further studies are needed to investigate the predictive value of LDL-C/HDL-C ratio in the development of carotid plaque components

Acknowledgments

We thank all of the participants for their cooperation in this study and the staff of the Xinjiang Key Laboratory of Cardiovascular Disease Research for their dedicated assistance in sample collection.

Contributions

Y-N Y, QZ and FL conceived and designed the study. QZ, Y-H W, H-M L, and J-Y L performed the experiments and collected the data. Y-N Y, Y-T M and X-M L completed the quality of data. QZ and FL performed the statistical analysis and wrote the paper. All authors read and approved the final manuscript.

For peer review only - http://bmjopen.bmj.com/site/about/guidelines.xhtml

Funding: This work was supported by the National Natural Science Foundation of China (No. 81460069, 81700315, U1503322), Science and Technology Agency of Xinjiang Uygur Autonomous Region (No. 2016E02072), and Postgraduate Scientific Research Project of Xinjiang Uygur Autonomous Region (No. XJGRI2017081).

Competing interests: None declared.

Ethics approval: The study was carried out in accordance with the declaration of Helsinki and the study protocol was approved by the Ethical Committee of First Affiliated Hospital of Xinjiang Medical University

Data sharing statement: No additional data are available.

Figure legends:

Fig 1. Comparison of LDL-C/HDL-C ratio between groups

The level of LDL-C/HDL-C ratio in CAP group was significantly higher than non-CAP group ($P < 0.001$).

Fig 2. ROC curve analysis of LDL-C/HDL-C ratio for predicting CCAP

The area under the curve (AUC) of LDL-C/HDL-C ratio was 0.748 (95 % CI: 0.720 - 0.776, $P < 0.001$).

Reference

1. Popkin BM. Is the obesity epidemic a national security issue around the globe? *Curr Opin Endocrinol Diabetes Obes* 2011;18(5):328-31. doi: 10.1097/MED.0b013e3283471c74

2. Wang Y, Mi J, Shan XY, et al. Is China facing an obesity epidemic and the consequences? The trends in obesity and chronic disease in China. *Int J Obes (Lond)* 2007;31(1):177-88. doi: 10.1038/sj.ijo.0803354

3. Guh DP, Zhang W, Bansback N, et al. The incidence of co-morbidities related to obesity and overweight: a systematic review and meta-analysis. *BMC Public Health* 2009;9:88. doi: 10.1186/1471-2458-9-88

4. Prospective Studies C, Whitlock G, Lewington S, et al. Body-mass index and cause-specific mortality in 900 000 adults: collaborative analyses of 57 prospective studies. *Lancet* 2009;373(9669):1083-96. doi: 10.1016/S0140-6736(09)60318-4

5. Balakumar P, Maung UK, Jagadeesh G. Prevalence and prevention of cardiovascular disease and diabetes mellitus. *Pharmacol Res* 2016;113(Pt A):600-09. doi: 10.1016/j.phrs.2016.09.040

For peer review only - http://bmjopen.bmj.com/site/about/guidelines.xhtml

6. Caleyachetty R, Thomas GN, Toulis KA, et al. Metabolically Healthy Obese and Incident Cardiovascular Disease Events Among 3.5 Million Men and Women. *J Am Coll Cardiol* 2017;70(12):1429-37. doi: 10.1016/j.jacc.2017.07.763

7. Global BMIMC, Di Angelantonio E, Bhupathiraju Sh N, et al. Body-mass index and all-cause mortality: individual-participant-data meta-analysis of 239 prospective studies in four continents. *Lancet* 2016;388(10046):776-86. doi: 10.1016/S0140-6736(16)30175-1

8. Lim S, Despres JP, Koh KK. Prevention of atherosclerosis in overweight/obese patients. - In need of novel multi-targeted approaches. *Circ J* 2011;75(5):1019-27.

9. Ceponiene I, Klumbiene J, Tamuleviciute-Prasciene E, et al. Associations between risk factors in childhood (12-13 years) and adulthood (48-49 years) and subclinical atherosclerosis: the Kaunas Cardiovascular Risk Cohort Study. *BMC Cardiovasc Disord* 2015;15:89. doi: 10.1186/s12872-015-0087-0

10. Greenland P, Alpert JS, Beller GA, et al. 2010 ACCF/AHA guideline for assessment of cardiovascular risk in asymptomatic adults: a report of the American College of Cardiology Foundation/American Heart Association Task Force on Practice Guidelines. *J Am Coll Cardiol* 2010;56(25):e50-103. doi: 10.1016/j.jacc.2010.09.001

11. Lee S, Cho GY, Kim HS, et al. Common carotid intima-media thickness as a risk factor for outcomes in Asian patients with acute ST-elevation myocardial infarction. *Can J Cardiol* 2014;30(12):1620-6. doi: 10.1016/j.cjca.2014.06.026

12. Nambi V, Chambless L, He M, et al. Common carotid artery intima-media thickness is as good as carotid intima-media thickness of all carotid artery segments in improving prediction of coronary heart disease risk in the Atherosclerosis Risk in Communities (ARIC) study. *Eur Heart J* 2012;33(2):183-90. doi: 10.1093/eurheartj/ehr192

13. Gardin JM, Bartz TM, Polak JF, et al. What do carotid intima-media thickness and plaque add to the prediction of stroke and cardiovascular disease risk in older adults? The cardiovascular health study. *J Am Soc Echocardiogr* 2014;27(9):998-1005 e2. doi: 10.1016/j.echo.2014.06.013

14. Park HW, Kim WH, Kim KH, et al. Carotid plaque is associated with increased cardiac mortality in patients with coronary artery disease. *Int J Cardiol* 2013;166(3):658-63. doi: 10.1016/j.ijcard.2011.11.084

15. Cox AJ, Hsu FC, Agarwal S, et al. Prediction of mortality using a multi-bed vascular calcification score in the Diabetes Heart Study. *Cardiovasc Diabetol* 2014;13:160. doi: 10.1186/s12933-014-0160-5

16. Guo X, Li Z, Guo L, et al. An update on overweight and obesity in rural Northeast China: from lifestyle risk factors to cardiometabolic comorbidities. *BMC Public Health* 2014;14:1046. doi: 10.1186/1471-2458-14-1046

17. Sangros FJ, Torrecilla J, Giraldez-Garcia C, et al. Association of General and Abdominal Obesity With Hypertension, Dyslipidemia and Prediabetes in the PREDAPS Study. *Rev Esp Cardiol (Engl Ed)* 2017 doi: 10.1016/j.rec.2017.04.035

18. Skinner AC, Perrin EM, Moss LA, et al. Cardiometabolic Risks and Severity of Obesity in Children and Young Adults. *N Engl J Med* 2015;373(14):1307-17. doi: 10.1056/NEJMoa1502821

19. Cai L, Zhang L, Liu A, et al. Prevalence, awareness, treatment, and control of dyslipidemia among adults in Beijing, China. *J Atheroscler Thromb* 2012;19(2):159-68.

20. Nishikido T, Oyama J, Keida T, et al. High-dose statin therapy with rosuvastatin reduces small dense LDL and MDA-LDL: The Standard versus high-dose therApy with Rosuvastatin for lipiD lowering (SARD) trial. *J Cardiol* 2016;67(4):340-6. doi: 10.1016/j.jjcc.2015.05.017

21. Valensi P, Avignon A, Sultan A, et al. Atherogenic dyslipidemia and risk of silent coronary artery disease in asymptomatic patients with type 2 diabetes: a cross-sectional study. *Cardiovasc Diabetol* 2016;15(1):104.

For peer review only - http://bmjopen.bmj.com/site/about/guidelines.xhtml

doi: 10.1186/s12933-016-0415-4

22. Enomoto M, Adachi H, Hirai Y, et al. LDL-C/HDL-C Ratio Predicts Carotid Intima-Media Thickness Progression Better Than HDL-C or LDL-C Alone. *J Lipids* 2011;2011:549137. doi: 10.1155/2011/549137

23. Katakami N, Kaneto H, Osonoi T, et al. Usefulness of lipoprotein ratios in assessing carotid atherosclerosis in Japanese type 2 diabetic patients. *Atherosclerosis* 2011;214(2):442-7. doi: 10.1016/j.atherosclerosis.2010.10.035

24. Ding D, Li X, Qiu J, et al. Serum lipids, apolipoproteins, and mortality among coronary artery disease patients. *Biomed Res Int* 2014;2014:709756. doi: 10.1155/2014/709756

25. Kunutsor SK, Zaccardi F, Karppi J, et al. Is High Serum LDL/HDL Cholesterol Ratio an Emerging Risk Factor for Sudden Cardiac Death? Findings from the KIHD Study. *J Atheroscler Thromb* 2017;24(6):600-08. doi: 10.5551/jat.37184

26. James PA, Oparil S, Carter BL, et al. 2014 evidence-based guideline for the management of high blood pressure in adults: report from the panel members appointed to the Eighth Joint National Committee (JNC 8). *JAMA* 2014;311(5):507-20. doi: 10.1001/jama.2013.284427

27. Polak JF, Pencina MJ, Pencina KM, et al. Carotid-wall intima-media thickness and cardiovascular events. *N Engl J Med* 2011;365(3):213-21. doi: 10.1056/NEJMoa1012592

28. National Cholesterol Education Program Expert Panel on Detection E, Treatment of High Blood Cholesterol in A. Third Report of the National Cholesterol Education Program (NCEP) Expert Panel on Detection, Evaluation, and Treatment of High Blood Cholesterol in Adults (Adult Treatment Panel III) final report. *Circulation* 2002;106(25):3143-421.

29. Fernandez-Friera L, Penalvo JL, Fernandez-Ortiz A, et al. Prevalence, Vascular Distribution, and Multiterritorial Extent of Subclinical Atherosclerosis in a Middle-Aged Cohort: The PESA (Progression of Early Subclinical Atherosclerosis) Study. *Circulation* 2015;131(24):2104-13. doi: 10.1161/CIRCULATIONAHA.114.014310

30. Singh GM, Danaei G, Farzadfar F, et al. The age-specific quantitative effects of metabolic risk factors on cardiovascular diseases and diabetes: a pooled analysis. *PLoS One* 2013;8(7):e65174. doi: 10.1371/journal.pone.0065174

31. Emerging Risk Factors C, Wormser D, Kaptoge S, et al. Separate and combined associations of body-mass index and abdominal adiposity with cardiovascular disease: collaborative analysis of 58 prospective studies. *Lancet* 2011;377(9771):1085-95. doi: 10.1016/S0140-6736(11)60105-0

32. Kim TJ, Shin HY, Chang Y, et al. Metabolically healthy obesity and the risk for subclinical atherosclerosis. *Atherosclerosis* 2017;262:191-97. doi: 10.1016/j.atherosclerosis.2017.03.035

33. Mancusi C, Gerdts E, Losi MA, et al. Differential effect of obesity on prevalence of cardiac and carotid target organ damage in hypertension (the Campania Salute Network). *Int J Cardiol* 2017;244:260-64. doi: 10.1016/j.ijcard.2017.06.045

34. Rovella V, Anemona L, Cardellini M, et al. The role of obesity in carotid plaque instability: interaction with age, gender, and cardiovascular risk factors. *Cardiovasc Diabetol* 2018;17(1):46. doi: 10.1186/s12933-018-0685-0

35. Rosvall M, Persson M, Ostling G, et al. Risk factors for the progression of carotid intima-media thickness over a 16-year follow-up period: the Malmo Diet and Cancer Study. *Atherosclerosis* 2015;239(2):615-21. doi: 10.1016/j.atherosclerosis.2015.01.030

36. Sandfort V, Lai S, Ahlman MA, et al. Obesity Is Associated With Progression of Atherosclerosis During Statin Treatment. *J Am Heart Assoc* 2016;5(7) doi: 10.1161/JAHA.116.003621

37. Manninen V, Tenkanen L, Koskinen P, et al. Joint effects of serum triglyceride and LDL cholesterol and

For peer review only - http://bmjopen.bmj.com/site/about/guidelines.xhtml

HDL cholesterol concentrations on coronary heart disease risk in the Helsinki Heart Study. Implications for treatment. *Circulation* 1992;85(1):37-45.

38. Hodis HN, Mack WJ, Azen SP, et al. Triglyceride- and cholesterol-rich lipoproteins have a differential effect on mild/moderate and severe lesion progression as assessed by quantitative coronary angiography in a controlled trial of lovastatin. *Circulation* 1994;90(1):42-9.

39. Wu TW, Hung CL, Liu CC, et al. Associations of Cardiovascular Risk Factors with Carotid Intima-Media Thickness in Middle-Age Adults and Elders. *J Atheroscler Thromb* 2017;24(7):677-86. doi: 10.5551/jat.37895

40. Nimkuntod P, Tongdee P. Plasma Low-Density Lipoprotein Cholesterol/High-Density Lipoprotein Cholesterol Concentration Ratio and Early Marker of Carotid Artery Atherosclerosis. *J Med Assoc Thai* 2015;98 Suppl 4:S58-63.

41. Tian L, Liu Y, Qin Y, et al. Association of the low-density lipoprotein cholesterol/high-density lipoprotein cholesterol ratio and concentrations of plasma lipids with high-density lipoprotein subclass distribution in the Chinese population. *Lipids Health Dis* 2010;9:69. doi: 10.1186/1476-511X-9-69

42. Chen QJ, Lai HM, Chen BD, et al. Appropriate LDL-C-to-HDL-C Ratio Cutoffs for Categorization of Cardiovascular Disease Risk Factors among Uygur Adults in Xinjiang, China. *Int J Environ Res Public Health* 2016;13(2):235. doi: 10.3390/ijerph13020235

43. Kurabayashi M, Yamazaki T, Group SS. Superior benefit of aggressive lipid-lowering therapy for high-risk patients using statins: the SUBARU study--more hypercholesterolemic patients achieve Japan Atherosclerosis Society LDL-C goals with rosuvastatin therapy than with atorvastatin therapy. *J Atheroscler Thromb* 2008;15(6):314-23.

44. Nicholls SJ, Tuzcu EM, Sipahi I, et al. Statins, high-density lipoprotein cholesterol, and regression of coronary atherosclerosis. *JAMA* 2007;297(5):499-508. doi: 10.1001/jama.297.5.499

For peer review only

For peer review only - http://bmjopen.bmj.com/site/about/guidelines.xhtml

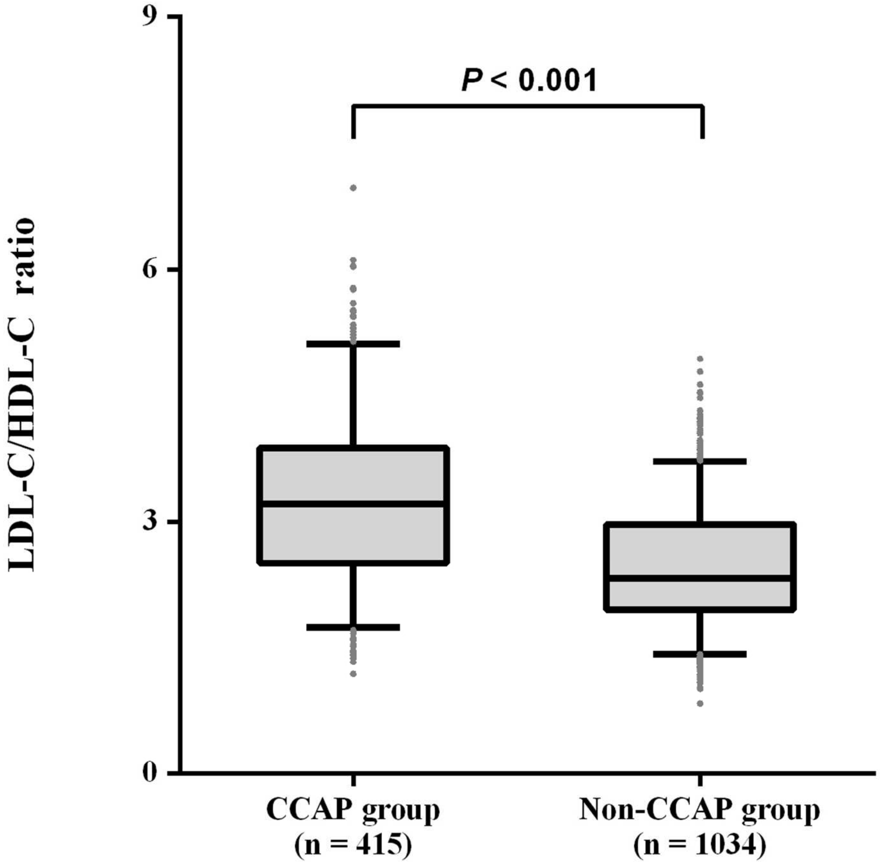

Fig 1. Comparison of LDL-C/HDL-C ratio between groups
The level of LDL-C/HDL-C ratio in CAP group was significantly higher than non-CAP group (P < 0.001).

111x112mm (300 x 300 DPI)

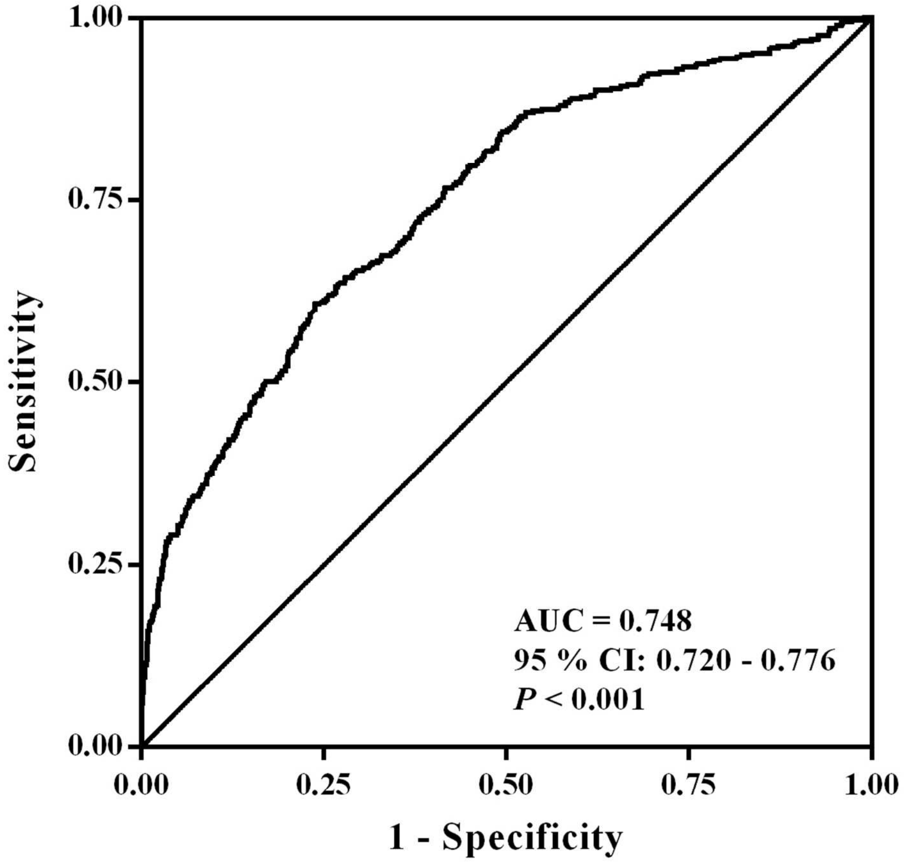

Fig 2. ROC curve analysis of LDL-C/HDL-C ratio for predicting CCAP
The area under the curve (AUC) of LDL-C/HDL-C ratio was 0.748 (95 % CI: 0.720 - 0.776, P < 0.001).

109x102mm (300 x 300 DPI)

STROBE Statement

Checklist of items that should be included in reports of observational studies

Section/Topic	Item No	Recommendation	Reported on Page No
**Title and abstract**	1	(*a*) Indicate the study's design with a commonly used term in the title or the abstract	1-2
		(*b*) Provide in the abstract an informative and balanced summary of what was done and what was found	2
**Introduction**			
Background/rationale	2	Explain the scientific background and rationale for the investigation being reported	4-5
Objectives	3	State specific objectives, including any prespecified hypotheses	5
**Methods**			
Study design	4	Present key elements of study design early in the paper	5
Setting	5	Describe the setting, locations, and relevant dates, including periods of recruitment, exposure, follow-up, and data collection	5
Participants	6	(*a*) *Cohort study*—Give the eligibility criteria, and the sources and methods of selection of participants. Describe methods of follow-up<br>*Case-control study*—Give the eligibility criteria, and the sources and methods of case ascertainment and control selection. Give the rationale for the choice of cases and controls<br>*Cross-sectional study*—Give the eligibility criteria, and the sources and methods of selection of participants	5-6
		(*b*) *Cohort study*—For matched studies, give matching criteria and number of exposed and unexposed<br>*Case-control study*—For matched studies, give matching criteria and the number of controls per case	
Variables	7	Clearly define all outcomes, exposures, predictors, potential confounders, and effect modifiers. Give diagnostic criteria, if applicable	5-6
Data sources/measurement	8*	For each variable of interest, give sources of data and details of methods of assessment (measurement). Describe comparability of assessment methods if there is more than one group	5-6
Bias	9	Describe any efforts to address potential sources of bias	5-6
Study size	10	Explain how the study size was arrived at	6
Quantitative variables	11	Explain how quantitative variables were handled in the analyses. If applicable, describe which groupings were chosen and why	7
Statistical methods	12	(*a*) Describe all statistical methods, including those used to control for confounding	7
		(*b*) Describe any methods used to examine subgroups and interactions	7
		(*c*) Explain how missing data were addressed	
		(*d*) *Cohort study*—If applicable, explain how loss to follow-up was addressed<br>*Case-control study*—If applicable, explain how matching of cases and controls was addressed<br>*Cross-sectional study*—If applicable, describe analytical methods taking account of sampling strategy	7
		(*e*) Describe any sensitivity analyses	7

For peer review only - http://bmjopen.bmj.com/site/about/guidelines.xhtml

Section/Topic	Item No	Recommendation	Reported on Page No
**Results**			
Participants	13*	(a) Report numbers of individuals at each stage of study—eg numbers potentially eligible, examined for eligibility, confirmed eligible, included in the study, completing follow-up, and analysed	7
		(b) Give reasons for non-participation at each stage	
		(c) Consider use of a flow diagram	
Descriptive data	14*	(a) Give characteristics of study participants (eg demographic, clinical, social) and information on exposures and potential confounders	7-8
		(b) Indicate number of participants with missing data for each variable of interest	
		(c) *Cohort study*—Summarise follow-up time (eg, average and total amount)	
Outcome data	15*	*Cohort study*—Report numbers of outcome events or summary measures over time	
		Case-control study—Report numbers in each exposure category, or summary measures of exposure	
		Cross-sectional study—Report numbers of outcome events or summary measures	7
Main results	16	(*a*) Give unadjusted estimates and, if applicable, confounder-adjusted estimates and their precision (eg, 95% confidence interval). Make clear which confounders were adjusted for and why they were included	8-12
		(*b*) Report category boundaries when continuous variables were categorized	11
		(*c*) If relevant, consider translating estimates of relative risk into absolute risk for a meaningful time period	
Other analyses	17	Report other analyses done—eg analyses of subgroups and interactions, and sensitivity analyses	11
**Discussion**			
Key results	18	Summarise key results with reference to study objectives	14
Limitations	19	Discuss limitations of the study, taking into account sources of potential bias or imprecision. Discuss both direction and magnitude of any potential bias	14-17
Interpretation	20	Give a cautious overall interpretation of results considering objectives, limitations, multiplicity of analyses, results from similar studies, and other relevant evidence	15-16
Generalisability	21	Discuss the generalisability (external validity) of the study results	17
**Other Information**			
Funding	22	Give the source of funding and the role of the funders for the present study and, if applicable, for the original study on which the present article is based	18

**Give information separately for cases and controls in case-control studies and, if applicable, for exposed and unexposed groups in cohort and cross-sectional studies.*

Note: An Explanation and Elaboration article discusses each checklist item and gives methodological background and published examples of transparent reporting. The STROBE checklist is best used in conjunction with this article (freely available on the Web sites of PLoS Medicine at http://www.plosmedicine.org/, Annals of Internal Medicine at http://www.annals.org/, and Epidemiology at http://www.epidem.com/). Information on the STROBE Initiative is available at www.strobe-statement.org.

For peer review only - http://bmjopen.bmj.com/site/about/guidelines.xhtml

For peer review only
